# Physical activity and anthropometric factors as predictors for postural stability in children

**DOI:** 10.1038/s41598-026-55265-7

**Published:** 2026-05-27

**Authors:** Saskia Brummer, Simon Flock, Anna-Marie Berelsmann, Martin Scholten, Christian Dobel, Orlando Guntinas-Lichius

**Affiliations:** 1 Department of Otorhinolaryngology, Jena University Hospital, Friedrich Schiller University Jena, Jena, Germany; 2 Department of Pediatrics and Adolescent Medicine, Jena University Hospital, Friedrich Schiller University Jena, Jena, Germany

**Keywords:** Balance, Obesity, Overweight, Activity, Posturography, Sports, Static stability, Health care, Medical research, Risk factors

## Abstract

To determine whether anthropometric factors and different domains of physical activity are associated with postural stability outcomes in children. Ninety-five children aged 7–18 years were enrolled in a cross-sectional observational study. Exposures included demographic variables, BMI category (according to German percentiles), physical education (PE), and factor scores for physical activity domains derived from exploratory factor analysis. Postural stability was assessed using computerized dynamic posturography, including scores of Sensory Organization Test (SOT), Limits of Stability (LOS), and Motor Control Test (MCT). Primary hypotheses about BMI, height, sex, and PE grades were pre-specified, and factor-analytic activity scores were added as predictors. Among 95 participants (mean [SD] age, 13.0 [2.6] years; 44 [46%] male), an exploratory factor analysis of 12 self-reported activity indicators identified five domains: daily activity, cycling, walking, sports club activity, and leisure-time activity. These domains explained 72% of the variance. Age, height, PE, weight, daily activity, and leisure-time activity were associated with postural stability measures. Older age correlated with better directional control (β = 0.37; 95% CI, 0.03–0.71; *P* = .03) and higher SOT scores (β = 0.35; 95% CI, 0.01–0.68; *P* = .04). Lower PE grades were related to poorer directional control (β = −0.24; 95% CI, − 0.47 to − 0.02; *P* = .03). Overweight was linked to lower endpoint excursion (β = −0.65; 95% CI, − 1.27 to − 0.02; *P* = .04), and obesity to less favorable balance strategies (β = −0.62; 95% CI, − 1.08 to − 0.15; *P* = .009). Sports club participation moderated the association between obesity and MCT latency (β = −0.65; 95% CI, − 1.08 to − 0.22; *P* = .004). Model fit was modest (adjusted R² = 0.04–0.23). Postural stability correlated with school PE grades and several physical activity domains. Findings highlight complex biological-behavioral interactions, and the need for multidimensional assessment of motor, and balance competencies in youth.

**Supplementary Information:**

The online version contains supplementary material available at 10.1038/s41598-026-55265-7.

## Introduction

Postural control refers to the ability to maintain the body’s center of gravity within the base of support to ensure stability.^1^ It encompasses postural orientation through sensory integration and postural equilibrium through coordinated movement strategies. These strategies help prevent falls during voluntary actions or external perturbations.^2,3^ Postural stability, defined as the maintenance of stability during quiet standing, represents a core component of postural control.^4^.

In children, postural stability is not yet fully matured due to ongoing motor development and limited muscular strength.^5–7^ This places them at increased risk for falls and sports-related injuries, which represent a leading cause of emergency visits and health care costs in this age group.^8–10^ In daily life, poor balance is associated with difficulties in fundamental motor tasks such as walking, stair negotiation, and object manipulation, which can limit independence and participation.^11^ In sports, insufficient neuromuscular control and balance increase the risk of acute injuries, particularly ankle sprains and knee ligament injuries, due to compromised joint stabilization. Over time, chronic deficits in postural control may contribute to maladaptive movement patterns and musculoskeletal misalignments, including altered lower limb biomechanics and potential progression of spinal deviations such as scoliosis.^12^ Furthermore, reduced balance ability in childhood has been linked to lower physical activity levels and poorer overall fitness, which may track into adulthood and elevate long-term health risks.^13^ Therefore, early identification and intervention targeting postural stability are critical for promoting safe participation in daily and athletic activities and preventing adverse health outcomes.

Several individual factors have been linked to balance performance. Higher body mass index (BMI) has been associated with poorer stability in children.^14,15^ Evidence on sex and height differences is mixed.^16–18^ Physical activity is consistently related to balance in adults,^19,20^ but evidence in children is limited. In the school setting, physical education (PE) grades provide an integrated assessment of endurance, coordination, agility, and strength and may serve as a pragmatic proxy for overall motor ability.^21^.

Despite these indications, research systematically addressing multiple predictors of postural stability in children is limited. Previous research has primarily investigated isolated determinants of postural stability in children, such as age, anthropometric characteristics, or sensory input.^13,22^ Age-related improvements in balance have been consistently reported, reflecting maturation of sensory–motor integration,^23,24^ while anthropometric factors such as body mass index have been associated with postural sway.^25,26^ In parallel, studies focusing on sensory contributions have demonstrated the critical role of visual, vestibular, and proprioceptive inputs in maintaining balance.^27,28^ However, few studies have simultaneously examined multiple domains—including biological, behavioral, and sensory factors—within a single analytical framework. Most investigations are limited to a small set of predictors, such as age and body composition or isolated sensory manipulations.^29,30^ Moreover, existing multivariate models often demonstrate low explanatory power, indicating that a substantial proportion of variance in postural stability remains unexplained.^31,32^ This suggests that the interaction between determinants—such as anthropometry, physical activity, and sensory integration—remains insufficiently understood. Therefore, the present study investigated BMI, body height, sex, physical education (PE) grades, and physical activity factors as potential predictors of postural stability in children using regression-based analyses.

## Methods

This cross-sectional observational study was conducted at the Jena University Hospital, Jena, Germany, between August 2023 and May 2024. Participants were children and adolescents aged 7–17 years and were recruited into three weight categories defined according to sex-specific percentiles by Kromeyer-Hauschild.^33^ Children and adolescents with a BMI > 90th percentile were considered overweight, and those with a BMI > 97th percentile were considered obese. A BMI between the 10th and 90th sex-specific percentiles was considered normal weight.^33^ Those with overweight or obesity were recruited via the pediatric endocrinology outpatient clinic. For the control group, recruitment was carried out via notices in sports clubs, pediatricians’ offices, and on the internal intranet of Jena University Hospital.

The exclusion criteria for all children were known peripheral or central vestibular disorders, acute musculoskeletal and neuromuscular diseases, degenerative neurological diseases, and the use of anti-vertigo medication. In addition, children with a BMI < 10th percentile (defined as underweight) were also excluded.

Participation was voluntary with a compensation of 30 euros. Written informed consent was obtained from participants and guardians. The protocol was approved by the local ethics committee (ethics committee of the Jena University Hospital, Jena, Germany, approval no.: 2023-3063-BO). All methods were performed in accordance with the relevant guidelines and regulations. Sample size adequacy varied across models according to model complexity. For the simplest regression models, the available sample exceeded the minimum required sample size, whereas the most complex model required 127 participants under conservative assumptions (R^2^ = 0.13, α = 0.05, power = 0.80).

### Workflow of the assessments

All examinations were conducted in the posturography examination room at the Department of Otorhinolaryngology, Jena University Hospital, Jena, Germany. After verifying the inclusion and exclusion criteria, participants and their legal guardians received information about the study. Written consent was obtained, and participants’ physical characteristics (age, height, weight, BMI) were recorded. The participants then completed the MoMo-AFB physical activity questionnaire^34^ on their own with their parents’ help. If there were any questions or illogical entries, we asked for clarification and adjusted the answer accordingly. Finally, the posturography tests were administered without shoes. Each participant was secured to the device using a vest and a fixed harness system, so that falling was not possible. Fifteen minutes were allocated for completing the questionnaire. The posturography tests took 25 min to complete. This resulted in a total duration of approximately 40 min per participant.

### Postural stability assessment

Postural stability was assessed using a computerized dynamic posturography platform (NeuroCom^®^ SMART EquiTest System (Natus Medical Incorporated, Pleasanton, USA). Three standardized test protocols were administered, including the Sensory Organization Test (SOT), the Limits of Stability (LOS), and the Motor Control Test (MCT). For the SOT, higher composite and equilibrium scores indicate better stability. Higher strategy scores indicate a greater reliance on ankle rather than hip-dominated strategies.^35,36^ The LOS test assesses voluntary postural control (reaction time, movement velocity, directional control, excursion).^37^ The MCT assesses automatic postural responses to unexpected perturbations.^38^ For all outcomes, higher values indicated better performance except for reaction time, where shorter times reflect superior performance. All variables of the postural stability assessment are listed in the **Supplementary Material S1**.

### Constitutional and predictor variables

Predictor variables included age (years), sex, and body height (cm). BMI was incorporated as a categorical variable using German sex-specific percentiles for normal weight, overweight, and obesity.^33^ All variables were derived from the MoMo Physical Activity Questionnaire (version 2016).^34^ This questionnaire was designed to measure the physical activity of children and adolescents. The reliability and validity of the MoMo-AFB is comparable with other international physical activity questionnaires for children and adolescents.^39^ The MoMo-AFB consists of 28 items. It covers everyday physical activity, sports within and outside of organized clubs, physical education, and compliance with physical activity guidelines. For each of these activity areas, duration and frequency are asked. In addition, for sports activities (school sports, club sports, and recreational sports), the perceived intensity and seasonality are also recorded.

The metabolic equivalent (MET) is an important measure in the questionnaire used to quantify the participants’ energy expenditure during physical activity. One MET describes the oxygen consumption of a sedentary adult and corresponds to approximately 3.5 ml of O₂ per kilogram of body weight per minute. During physical exertion, the MET increases in proportion to oxygen consumption.^40^ The activities listed in the questionnaire were categorized into MET values based on their energy requirements, thereby enabling the calculation of MET-hours per week. The most recent PE grade was reported by the participants and parents themselves. The PE grades, awarded in the German school system, are standardized nationwide by the government, and range from 1 (very good) to 6 (inadequate; failed)). The PE grade served as an indicator of motor performance. In addition, factor scores representing different aspects of physical activity were extracted from an exploratory factor analysis of the MoMo Physical Activity Questionnaire scores.

### Statistical analysis

All statistical analyses were performed using RStudio (Version 4.2.2).^41^ Exploratory factor analysis (EFA) with maximum likelihood estimation and oblimin rotation was conducted on 12 indicators of physical activity. Sampling adequacy was assessed with the Kaiser-Meyer-Olkin measure and Bartlett’s test. The number of factors was determined by parallel analysis, eigenvalues, and interpretability. Factor scores were computed using the regression method and used in subsequent analysis. Additional information on the results of model diagnostics can be found in **Supplementary Material S2** and **Supplementary Material S3**,** Supplementary Table ****S1****-S7**.

Elastic net regression with 10-fold cross-validation was employed for data-driven exploratory predictor selection. Elastic net regression was chosen because it combines variable selection with regularization, making it appropriate for datasets with multiple potentially correlated predictors. Results from the λmin solution are reported in the main text, while the more parsimonious λ1se solution is presented in **Supplementary Table S8**. Predictors identified via elastic net were subsequently entered into multiple linear regression models, controlling for age, sex, body height, BMI category, and PE grade. Continuous predictors were z-standardized to facilitate comparability. Elastic net regression models were specified, including both main effects and interaction terms of BMI categories with measures of physical activity. Model diagnostics included visual inspection of residual plots, tests for heteroscedasticity (Breusch-Pagan), assessment of normality (Q-Q plots), and multicollinearity (VIF). The corresponding results are shown in **Supplementary Figure ****S1**, **Supplementary Figure ****S2**, and **Supplementary Table S9**.

Finally, an exploratory path model was specified to integrate the main predictors of postural stability outcomes. The model included demographic covariates and activity-related factor scores identified in the previous analyses. Continuous physical activity variables and covariates were entered in their original metric to allow straightforward interpretation of main effects. For interaction terms with BMI categories, these continuous predictors were mean-centered before computing the product terms to reduce collinearity. The model was estimated using maximum likelihood, and model fit was evaluated with standard fit indices. Statistical significance was determined using a threshold of *P* < .05.

## Results

### Study population

**Fig. 1:**
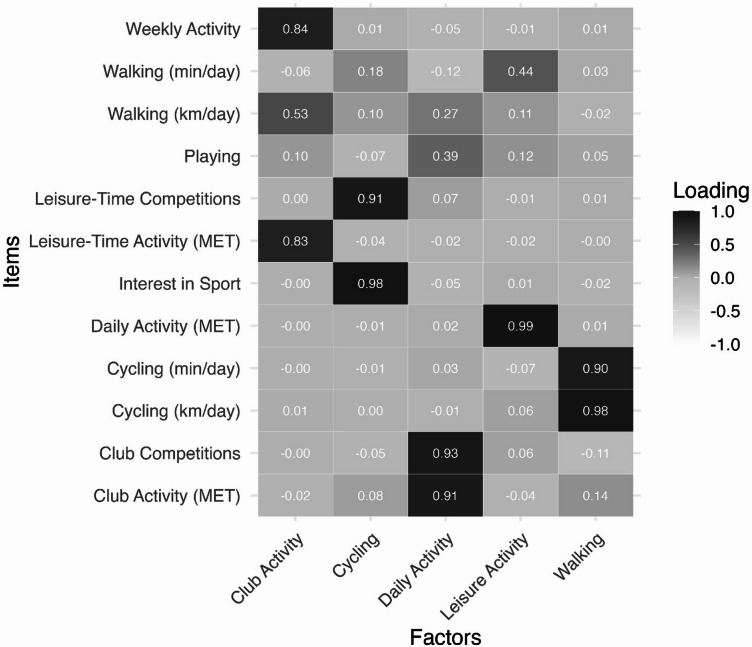
Heatmap of factor loadings from the exploratory analysis of physical activity indicators. The heatmap shows standardized factor loadings (range − 1 to 1) of 12 indicators across the five extracted factors. Darker shading indicates stronger positive loadings. MET, metabolic equivalent of task.

The study included 95 participants (mean [SD] age, 13.0 [2.6] years; 44 [46%] male and 51 [54%] female). Table 1 presents additional sample characteristics stratified by BMI category. EFA of 12 indicators yielded a five-factor solution, explaining 72% of the total variance (χ^2^(16) = 20.84; *P* = .18, RMSEA = 0.06 [90% CI, 0.00 − 0.12]; TLI = 0.96; RMSR = 0.02). Extracted factors were Daily Activity, Cycling Activity, Walking, Sports Club Activity, and Leisure-Time Activity. Corresponding descriptives and factor loadings are presented in the Supplement. Factor loadings of the exploratory factor analysis are illustrated in Fig. 1.

**Table 1:** Sample characteristics by BMI category.

	Overall (*n* = 95)^1^	Normal weight (*n* = 51)^1^	Overweight (*n* = 12)^1^	Obesity (*n* = 32)^1^
Age in years, mean (SD)	13.03 (2.64)	13.25 (2.79)	13.83 (2.52)	12.38 (2.34)
Female Sex, n (%)	51/95 (54%)	28/51 (55%)	8/12 (67%)	15/32 (47%)
Male Sex, n (%)	44/95 (46%)	23/51 (45%)	4/12 (33%)	17/32 (53%)
Height in cm, mean (SD)	161.19 (13.38)	160.19 (13.81)	163.77 (12.16)	161.81 (13.36)
Weight in kg, mean (SD)	64.32 (22.34)	50.99 (13.55)	67.45 (12.14)	84.39 (21.27)
BMI^a^, mean (SD)	24.28 (6.51)	19.43 (2.67)	24.93 (1.79)	31.77 (4.36)
Activity, mean (SD)				
Activity Daily (MET)^b^	47.16 (46.28)	39.42 (34.62)	56.67 (46.77)	55.93 (59.78)
Club Activity (MET)^b^	17.65 (26.97)	23.53 (30.15)	13.25 (27.29)	9.92 (18.68)
Leisure-Time Activity (MET)^b^	10.73 (22.11)	11.36 (23.61)	9.10 (13.38)	10.34 (22.75)
Physical Education Grade^c^	1.74 (1.08)	1.39 (0.98)	2.18 (0.58)	2.15 (1.19)

^1^Data are presented as mean (SD) for continuous variables and No./total No. (%) for categorical variables.

^a^BMI indicates body mass index; categories defined according to German reference percentiles for children.^23^

^b^MET indicates metabolic equivalent of task; values were derived from published activity classifications.^24c^German 6-point grading system ranging from 1 to 6. The lower the grade, the better it is: a “1” is an excellent grade, whereas “5 and “6” are fail grades.

### Exploratory predictor selection

To identify key factors influencing postural stability outcomes, elastic net regression modelling was performed, selecting predictors with the minimum lambda (λmin) value.

Body height showed positive coefficients for LOS directional control (0.10) and negative coefficients for LOS endpoint excursion (-0.18). Maximum excursion was most strongly associated with daily activity (–3.99) and PE grade (–3.08). For the MCT, age (–9.05) was the strongest predictor, with additional associations for walking (2.89) and leisure-time activity (2.48). For the SOT, body height showed positive coefficients for the composite score (0.28) and conditions E5 (0.36) and E6 (0.45). Relevant predictors in S5 included age (7.12), PE grade (-4.03), club activity (0.93), and obesity (-8.45). In S6, age (5.70), PE grade (-5.59), and obesity (-5.87) were most relevant. Interaction effects (e.g., PE grade x overweight; club activity x obesity), as well as the λ1se solution and full coefficient paths, are reported in the Supplement.

### Regression analyses for primary outcomes

Multiple linear regression models were estimated using predictors selected by the elastic net. Model diagnostics indicated no major violations of regression assumptions. Adjusted R^2^ values ranged from 0.04 to 0.23, indicating modest explained variance. Results are reported separately for each test used as an outcome, with additive models first, followed by the results of interaction models. The most important model parameters are reported in Table 2, and full model parameters, as well as model fit indices in **Supplementary Table S10** and **Supplementary Table 11**.

**Table 2:** Linear regression coefficients of models predicting postural stability outcomes.

Model	Predictor	Est (95% CI)	β (95% CI)	*P*
DCL Additive	Age (y)	3.90 (0.34, 7.47)	0.37 (0.03, 0.71)	0.032
	PE grade (z)	− 6.78 (− 12.99, − 0.57)	− 0.24 (− 0.47, − 0.02)	0.033
EPE Additive	Overweight	− 17.88 (− 35.10, − 0.66)	− 0.65 (− 1.27, − 0.02)	0.042
	PE grade (z)	− 5.81 (− 11.86, 0.24)	− 0.21 (− 0.43, 0.01)	0.060
MCT Additive	PE grade (z)	− 10.00 (− 20.91, 0.91)	− 0.21 (− 0.44, 0.02)	0.072
	Leisure activity	8.28 (− 2.52, 19.08)	0.17 (− 0.05, 0.39)	0.131
	Walking	− 7.73 (− 19.59, 4.14)	− 0.16 (− 0.40, 0.08)	0.199
MCT Interaction	Obesity × club activity	− 30.88 (− 51.39, − 10.38)	− 0.65 (− 1.08, − 0.22)	0.004
	Club activity	2.15 (− 10.11, 14.41)	0.05 (− 0.21, 0.30)	0.728
MVL Additive	PE grade (z)	− 3.97 (− 7.41, − 0.54)	− 0.26 (− 0.49, − 0.04)	0.024
MXE Additive	Age (y)	2.74 (− 0.97, 6.44)	0.24 (− 0.09, 0.57)	0.146
	Obesity	− 10.17 (− 24.46, 4.12)	− 0.34 (− 0.83, 0.14)	0.161
	Overweight	− 14.61 (− 32.78, 3.56)	− 0.49 (− 1.11, 0.12)	0.114
	Daily activity	− 6.58 (− 12.77, − 0.39)	− 0.22 (− 0.42, − 0.01)	0.038
RT Additive	Age (y)	1.65 (− 0.24, 3.55)	0.30 (− 0.04, 0.64)	0.086
	Overweight	− 5.70 (− 15.08, 3.68)	− 0.39 (− 1.04, 0.25)	0.230
	PE grade (z)	− 4.02 (− 7.31, − 0.72)	− 0.28 (− 0.50, − 0.05)	0.017
SOT Additive	Age (y)	3.59 (0.11, 7.07)	0.35 (0.01, 0.68)	0.044
	PE grade (z)	− 6.32 (− 12.38, − 0.25)	− 0.23 (− 0.45, − 0.01)	0.041
SOT E5 Additive	Age (y)	3.34 (− 0.13, 6.81)	0.32 (− 0.01, 0.66)	0.059
	PE grade (z)	− 5.21 (− 11.25, 0.83)	− 0.19 (− 0.41, 0.03)	0.090
SOT E6 Additive	Age (y)	3.50 (− 0.07, 7.06)	0.32 (− 0.01, 0.66)	0.054
	PE grade (z)	− 6.47 (− 12.68, − 0.26)	− 0.23 (− 0.45, − 0.01)	0.041
SOT S5 Additive	Age (y)	3.55 (0.18, 6.92)	0.33 (0.02, 0.65)	0.039
	Obesity	− 17.20 (− 30.09, − 4.32)	− 0.62 (− 1.08, − 0.15)	0.009
	Overweight	− 14.95 (− 31.55, 1.65)	− 0.53 (− 1.13, 0.06)	0.077
	Body height (cm)	− 0.64 (− 1.24, − 0.03)	− 0.31 (− 0.59, − 0.02)	0.039
	PE grade (z)	− 4.88 (− 10.84, 1.08)	− 0.17 (− 0.39, 0.04)	0.107
	Club activity	2.84 (− 2.68, 8.36)	0.10 (− 0.10, 0.30)	0.309
SOT S5 Interaction	Age (y)	2.87 (− 1.34, 7.08)	0.27 (− 0.13, 0.67)	0.181
	Body height (cm)	− 0.58 (− 1.14, − 0.03)	− 0.28 (− 0.55, − 0.01)	0.038
	Daily Activity	1.50 (− 0.41, 3.41)	0.05 (− 0.02, 0.12)	0.124
	Leisure Activity	− 0.98 (− 5.58, 3.61)	− 0.03 (− 0.20, 0.13)	0.675
	Obesity	− 17.87 (− 28.27, − 7.47)	− 0.64 (− 1.02, − 0.26)	<0.001
	Obesity × Daily Activity	− 4.77 (− 23.18, 13.65)	− 0.17 (− 0.82, 0.49)	0.612
	Obesity × Leisure Activity	6.89 (− 21.95, 35.72)	0.24 (− 0.78, 1.27)	0.640
SOT S6 Additive	Age (y)	3.28 (− 0.16, 6.73)	0.31 (− 0.02, 0.63)	0.061
	Obesity	− 17.11 (− 29.98, − 4.24)	− 0.61 (− 1.07, − 0.15)	0.010
	PE grade (z)	− 6.29 (− 12.20, − 0.37)	− 0.22 (− 0.43, − 0.01)	0.038
	Daily activity	− 1.09 (− 6.83, 4.66)	− 0.04 (− 0.24, 0.16)	0.708
SOT S6 Interaction	Age (y)	2.89 (− 1.38, 7.16)	0.27 (− 0.14, 0.68)	0.185
	Daily Activity	1.45 (− 0.93, 3.83)	0.05 (− 0.03, 0.13)	0.231
	Leisure Activity	− 1.12 (− 5.55, 3.32)	− 0.04 (− 0.20, 0.12)	0.622
	Obesity	− 17.21 (− 25.75, − 8.67)	− 0.61 (− 0.92, − 0.30)	<0.001
	Obesity x Leisure Activity	9.32 (− 10.13, 28.76)	0.32 (− 0.36, 1.01)	0.348
	Overweight	− 8.75 (− 31.18, 13.68)	− 0.31 (− 1.12, 0.50)	0.445
	Overweight x Daily Activity	− 19.78 (− 70.67, 31.11)	− 0.69 (− 2.49, 1.11)	0.446
	PE grade (z)	− 5.18 (− 14.08, 3.73)	− 0.18 (− 0.51, 0.14)	0.255
	Sex (female)	1.83 (− 14.69, 18.35)	0.07 (− 0.53, 0.66)	0.828

Unstandardized coefficients with 95% CIs, standardized coefficients (β), and P values are reported. Models include additive (main effects only) and selected interaction terms. All models were adjusted for age, sex, height, BMI category, and physical education grade. All variables were entered in their original units, except PE Grade, which was z-standardized before model estimation. Extracted factor scores were mean-centered prior to analysis. BMI category was included as a categorical predictor with three levels: Normal Weight, Overweight, and Obesity. Heteroscedasticity-consistent (HC3) standard errors were used in models where heteroscedasticity was detected.

DCL, Directional Control; EPE, Endpoint Excursion; MVL, Movement Velocity; MXE, Maximum Excursion; RT, Reaction Time; MCT, Motor Control Test Latency; SOT, Sensory Organization Test; SOT E5, Sensory Organization Test Equilibrium Score, Condition 5; SOT E6, Sensory Organization Test Equilibrium Score, Condition 6; SOT S5, Sensory Organization Test Strategy Score, Condition 5; SOT S6, Sensory Organization Test Strategy Score, Condition 6; PE, Physical Education. BMI categories (Normal Weight, Overweight, Obesity) were defined according to German reference percentiles.^23^

### Results of limits of stability

Higher age was significantly associated with better directional control (*P* = .032), and a worse PE grade was associated with lower directional control (*P* = .033). For endpoint Excursion, overweight was significantly associated with lower scores compared to normal-weight peers (*P* = .042). A worse PE grade was negatively associated with movement velocity (*P* = .024) and reaction time (*P* = .017). Higher daily activity was associated with lower maximum excursion (*P* = .038). Full statistical details are presented in Table 2 and **Supplementary Table S10**.

### Results of motor control test

In the additive model, no predictors reached statistical significance. Higher walking activity was non-significantly associated with lower latency. Higher leisure activity and a better PE grade showed nonsignificant associations with higher latency. In obese children, higher club activity was significantly associated with lower latency (*P* = .004), whereas in normal-weight children the association was close to zero.

### Results of sensory organization test

Within the additive models, age was positively associated with higher SOT composite scores. A poorer PE grade showed an inverse association (*P* = .041), with similar results appearing for condition E6. In SOT condition S5, obesity (*P* = .009) and greater body height (*P* = .039) were significantly associated with a more hip-dominated strategy. Older age was associated with a more ankle-dominated strategy (*P* = .039). Similar results for obesity and PE grade were examined in SOT condition S6.

In interaction models, obesity and greater body height remained significantly associated with a hip-dominated strategy in SOT condition S5. Physical activity factors showed only nonsignificant trends, though the direction of effects differed for BMI groups.

### Exploratory path model

**Fig. 2:**
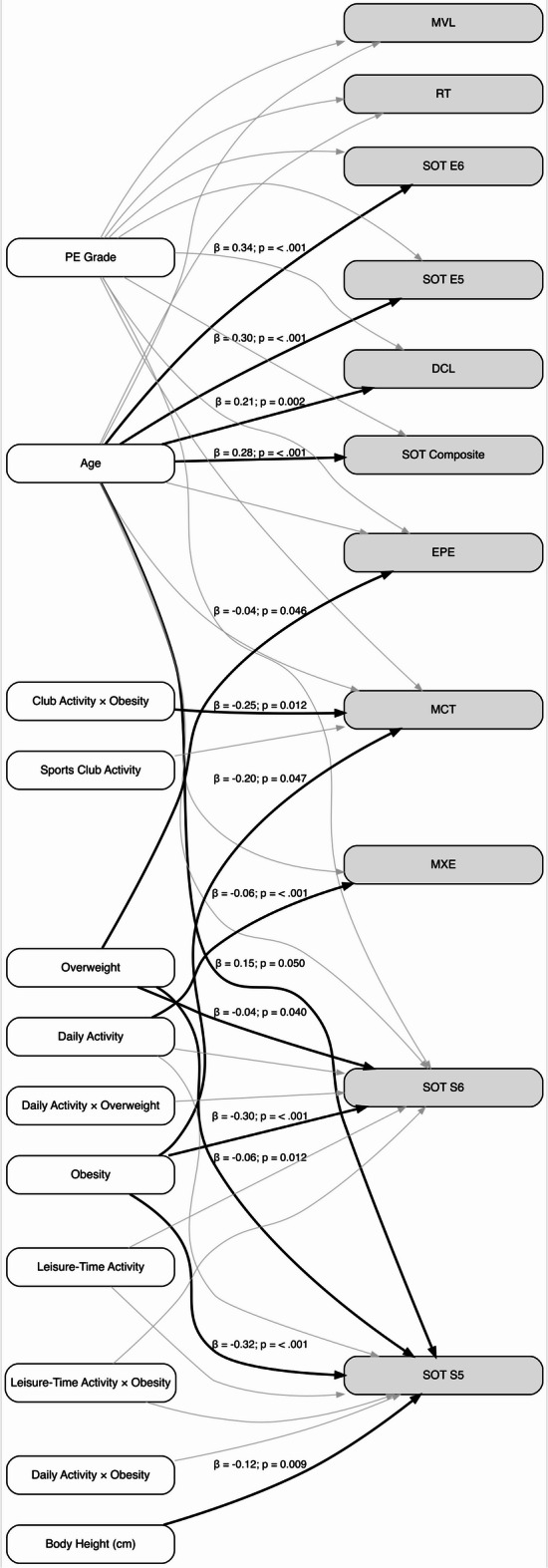
Exploratory structural path model. The model summarizes associations of demographic and activity-related predictors with postural stability outcomes. Solid black paths (*p*<.05) are shown with standardized coefficients. Thin gray paths represent non-significant associations (*p*≥.05, no labels). Outcomes are displayed in grey boxes, predictors in white boxes. LOS RT, Limits of Stability Reaction Time; LOS MVL, Limits of Stability Movement Velocity; LOS MXE, Limits of Stability Maximum Excursion; LOS DCL, Limits of Stability Directional Control; LOS EPE, Limits of Stability Endpoint Excursion; SOT, Sensory Organization Test; SOT E5, Sensory Organization Test Equilibrium Score 5; SOT E6, Sensory Organization Test Equilibrium Score 6; SOT S5, Sensory Organization Test Strategy Score 5; SOT S6, Sensory Organization Test Strategy Score 6; MCT, Motor Control Test latency.

An exploratory path model integrating key regression findings showed limited model fit, with some indices suggesting acceptable fit and others indicating poorer fit (CFI = 0.93, TLI = 0.85, RMSEA = 0.15, SRMR = 0.10). Given the mixed fit indices, the path model should be interpreted cautiously and viewed as exploratory rather than confirmatory. Age was positively associated with SOT Composite and LOS Directional Control. Higher daily activity was associated with lower Maximum Excursion. For MCT latency, obesity, and the interaction of club activity x obesity showed a significant negative association. Full estimates are presented in the Supplement. A schematic path diagram is shown in Fig. 2. All parameters and model fit indices are provided in **Supplementary Table S12** and **Supplementary Table 13**.

## Discussion

This study investigated physical activity in schools, clubs, and leisure time, alongside anthropometric factors, as predictors of postural stability in children. Key findings revealed associations of older age, worse PE grades, obesity, and obesity – activity interactions with poorer performance across multiple domains. Five physical activity factors (daily activity, cycling, walking, sports club activity, and leisure-time activity) were identified via exploratory factor analysis and used as predictors in subsequent models. Elastic net regression identified age, BMI category, body height, PE grade, and activity-related factors as consistent predictors of postural stability. Interactions between activity factors and BMI were associated with several outcomes. The exploratory path model largely converged with these findings.

Consistent with prior literature,^14,15^ higher BMI categories were associated with poorer stability across several outcomes. BMI thus appears to be an important predictor, although it does not capture the full complexity. In sports where balance is systematically trained, targeted practice may compensate for the disadvantages associated with higher body weight. This stresses the importance of considering both weight status and physical activity.

Another noteworthy finding was the interaction of obesity and greater body height with a more hip-dominated strategy, suggesting that BMI may have a particularly adverse influence on postural control in taller individuals.

With respect to physical activity, our results further indicate that not only the amount but also the type of activity plays a role in stability outcomes. Daily activity was inversely associated with maximum excursion. While no association was found in SOT S5 in normal-weight children, a negative but nonsignificant association emerged in obese children. Leisure-time activity showed no relation in normal-weight children but a nonsignificant positive association in those with obesity, with similar patterns in SOT S6. Thus, daily and leisure activity appear to load in opposite directions in higher BMI categories, while showing no association in normal-weight children. One possible explanation is that high levels of unsupervised daily activity may not provide the same functional adaptations as structured training and may therefore be associated with poorer balance outcomes.^42^ However, this interpretation remains speculative and requires further investigation. Notably, the SOT demonstrated increasing sensitivity with task complexity, as simpler conditions showed limited differentiation, while more challenging conditions yielded clearer associations, highlighting their superior discriminatory power.

Club activity was associated with lower MCT latency in obese children, whereas the association was near zero in normal-weight individuals. In SOT strategy scores, club activity showed only small, nonsignificant associations. These findings were unexpected, as sports club participation is generally associated with enhanced motor coordination.^43^ Their small effect sizes reflect the limited explanatory power of activity indicators derived from MET-based questionnaire classification, which capture quantity and intensity but not qualitative aspects of training (e.g., coordination, balance-specific tasks). This aligns with studies indicating that the type of sport influences balance abilities more strongly than overall activity level.^44,45^ Furthermore, not all sports clubs implement balance training,^46,47^ which may contribute to the low effect sizes.

In addition, developmental aspects should be considered. The duration and onset age of sports participation were not assessed, although both may influence stability development.^48,49^ The timing of activity exposure may be particularly important during sensitive developmental phases. Early activity trajectories, meaning the patterns of physical activity engagement and development during critical developmental periods, may shape the maturation of sensory and motor systems and influence postural control in later life.^50,51^ Understanding the timing, duration, and type of early activity is therefore crucial to clarify its impact on postural stability development.^50^.

One of the most consistent predictors across all models was PE grade. Because higher numerical values reflect poorer performance, the negative coefficients indicate that worse PE grades were consistently associated with lower postural stability. This suggests that this measure not only reflects general motor abilities, as described in previous work,^21^ but may also capture specific aspects of balance performance. It may therefore serve as a pragmatic indicator of children’s motor and stability development that other activity predictors did not capture. However, this interpretation should be made cautiously, as PE grades may vary between teachers and schools and therefore do not represent a fully objective measure of motor competence. Non-performance factors (motivation, social skills) influence PE grades. What is missing, are validated instruments to achieve better reliability.

This study has important limitations. The cross-sectional design precludes causal inference, so the observed associations cannot be interpreted as directional effects. Second, the relatively small sample size and the presence of interaction terms across several models may have limited statistical power, particularly for subgroup-specific effects. Although the available sample was sufficient for the simpler regression models, the more complex models with a larger number of predictors and interaction terms may have been underpowered, which should be considered when interpreting findings. In addition, the different recruitment pathways may have introduced selection bias, as children recruited through sports clubs may differ in physical activity level from the general population, despite mandatory school-based physical education in Germany. Physical activity was assessed by self-report using MET-based indicators, which may not reflect qualitative aspects of training. Effect sizes and explained variances were generally small, indicating that only a limited proportion of postural stability variance could be explained. Several potentially relevant variables were not assessed, including qualitative features of physical activity, balance-specific training, sport type, duration, and onset age of participation, as well as psychosocial (e.g., motivation, self-efficacy) and environmental factors. Given these limitations, the findings should be interpreted as exploratory and hypothesis-generating rather than confirmatory.

Future studies should address these limitations by using longitudinal or intervention designs, examining sport-specific training characteristics, and including more detailed measures of physical activity and developmental context. Sensitive developmental phases and biographical data on sports participation across the lifespan should also be considered, as early activity trajectories may critically shape later postural stability.

In summary, this study identified BMI category, PE grade, and selected physical activity domains as key predictors of postural stability in children, with evidence for BMI-specific moderation effects. While BMI was consistently associated with poorer balance, a better PE grade emerged as a pragmatic proxy for better motor and stability skills. Associations of activity factors varied by type and weight group.

These findings suggest that practical exercise recommendations should consider both BMI category and PE grade. Children with obesity or poorer PE grades may benefit from structured, balance-focused, moderate-intensity activities. Children with better postural stability may not require the same level of basic balance support. However, they may still benefit from progressively more challenging and sport-specific training to maintain and further develop their motor skills.

## Supplementary Information

Below is the link to the electronic supplementary material.

Supplementary Material 1

Supplementary Material 2

Supplementary Material 3

## Data Availability

The datasets used in the current study are available from the corresponding author on reasonable request.
