# Supplementary material for: Physical activity and anthropometric factors as predictors for postural stability in children
Source: Sci Rep. 2026 May 27;16:16425. doi: 10.1038/s41598-026-55265-7 (PMC13216329; doi:10.1038/s41598-026-55265-7)
Supplement: Supplementary file 1 — Supplementary Material 1 [file 41598_2026_55265_MOESM1_ESM.docx]

**Supplementary Material**

Saskia Brummer, Simon Flock, Anna-Marie Berelsmann, Martin Scholten, Christian Dobel, Orlando Guntinas-Lichius

**Physical activity and anthropometric factors as predictors for postural stability in children**

**Supplementary Methods S1.**

Description of original variables

**Supplementary Methods S2.**

Exploratory factor analysis

Elastic net regression

Linear regression models

Exploratory structural equation modelling

**Supplementary Methods S3.**

Linear regression models results

This supplemental material has been provided by the authors to give readers additional information about their work.

**Supplementary Methods S1**

**Description of original variables**

| Variable | Meaning |
| --- | --- |
| SOT Composite | SOT Composite Equilibrium Score |
| SOT_E1.1 | SOT Condition 1 Equilibrium trial 1 |
| SOT_E1.2 | SOT Condition 1 Equilibrium trial 2 |
| SOT_E1.3 | SOT Condition 1 Equilibrium trial 3 |
| SOT_E1_Com | SOT Condition 1 Equilibrium mean |
| SOT_S1.1 | SOT Condition 1 Strategy trial 1 |
| SOT_S1.2 | SOT Condition 1 Strategy trial 2 |
| SOT_S1.3 | SOT Condition 1 Strategy trial 3 |
| SOT_S1_Com | SOT Condition 1 Strategy mean |
| SOT_Fro1.1 | SOT Condition 1 COG frontal plane trial 1 |
| SOT_Fro1.2 | SOT Condition 1 COG frontal plane trial 2 |
| SOT_Fro1.3 | SOT Condition 1 COG frontal plane trial 3 |
| SOT_COG1_Fro_Com | SOT Condition 1 COG frontal plane mean |
| SOT_Sag1.1 | SOT Condtion 1 COG sagital trial 1 |
| SOT_Sag1.2 | SOT Condition 1 COG sagital trial 2 |
| SOT_Sag1.3 | SOT Condition 1 COG sagital trial 3 |
| SOT_COG1_Sag_Com | SOT Condition 1 COG Sagittal plane Mean |
| SOT_E2.1 | SOT Condition 2 Equilibrium Trial 1 |
| SOT_E2.2 | SOT Condition 2 Equilibrium Trial 2 |
| SOT_E2.3 | SOT Condition 2 Equilibrium Trial 3 |
| SOT_E2_Com | SOT Condition 2 Equilibirum Mean |
| SOT_S2.1 | SOT Condition 2 Strategy Trial 1 |
| SOT_S2.2 | SOT Condition 2 Strategy Trial 2 |
| SOT_S2.3 | SOT Condition 2 Strategy Trial 3 |
| SOT_S2_Com | SOT Condition 2 Strategy Mean |
| SOT_Fro2.1 | SOT Condition 2 COG Frontal plane Trial 1 |
| SOT_Fro2.2 | SOT Condition 2 COG Frontal plane Trial 2 |
| SOT_Fro2.3 | SOT Condition 2 COG Frontal plane Trial 3 |
| SOT_COG2_Fro_Com | SOT Condition 2 COG Frontal plane Mean |
| SOT_Sag2.1 | SOT Condtion 2 COG Sagittal plane Trial 1 |
| SOT_Sag2.2 | SOT Condition 2 COG Sagittal plane Trial 2 |
| SOT_Sag2.3 | SOT Condition 2 COG Sagittal plane Trial 3 |
| SOT_COG2_Sag_Com | SOT Condition 2 COG Sagittal plane Mean |
| SOT_E3.1 | SOT Condition 3 Equilibrium Trial 1 |
| SOT_E3.2 | SOT Condition 3 Equilibrium Trial 2 |
| SOT_E3.3 | SOT Condition 3 Equilibrium Trial 3 |
| SOT_E3_Com | SOT Condition 3 Equilibirium Mean |
| SOT_S3.1 | SOT Condition 3 Strategy Trial 1 |
| SOT_S3.2 | SOT Condition 3 Strategy Trial 2 |
| SOT_3.3 | SOT Condition 3 Strategy Trial 3 |
| SOT_S3_Com | SOT Condition 3 Strategy Mean |
| SOT_Fro3.1 | SOT Condition 3 COG Frontal plane Trial 1 |
| SOT_Fro3.2 | SOT Condition 3 COG Frontal plane Trial 2 |
| SOT_Fro3.3 | SOT Condition 3 COG Frontal plane Trial 3 |
| SOT_COG3_Fro_Com | SOT Condition 3 COG Frontal plane Mean |
| SOT_Sag3.1 | SOT Condtion 3 COG Sagittal plane Trial 1 |
| SOT_Sag3.2 | SOT Condition 3 COG Sagittal plane Trial 2 |
| SOT_Sag3.3 | SOT Condition 3 COG Sagittal plane Trial 3 |
| SOT_COG3_Sag_Com | SOT Condition 3 COG Sagittal plane Mean |
| SOT_E4.1 | SOT Condition 4 Equilibrium Trial 1 |
| SOT_E4.2 | SOT Condition 4 Equilibrium Trial 2 |
| SOT_E4.3 | SOT Condition 4 Equilibrium Trial 3 |
| SOT_E4_Com | SOT Condition 4 Equilibirum Mean |
| SOT_S4.1 | SOT Condition 4 Strategy Trial 1 |
| SOT_S4.2 | SOT Condition 4 Strategy Trial 2 |
| SOT_S4.3 | SOT Condition 4 Strategy Trial 3 |
| SOT_S4_Com | SOT Condition 4 Strategy Mean |
| SOT_Fro4.1 | SOT Condition 4 COG Frontal plane Trial 1 |
| SOT_Fro4.2 | SOT Condition 4 COG Frontal plane Trial 2 |
| SOT_Fro4.3 | SOT Condition 4 COG Frontal plane Trial 3 |
| SOT_COG4_Fro_Com | SOT Condition 4 COG Frontal plane Mean |
| SOT_Sag4.1 | SOT Condtion 4 COG Sagittal plane Trial 1 |
| SOT_Sag4.2 | SOT Condition 4 COG Sagittal plane Trial 2 |
| SOT_Sag4.3 | SOT Condition 4 COG Sagittal plane Trial 3 |
| SOT_COG4_Sag_Com | SOT Condition 4 COG Sagittal plane Mean |
| SOT_E5.1 | SOT Condition 5 Equilibrium Trial 1 |
| SOT_E5.2 | SOT Condition 5 Equilibrium Trial 2 |
| SOT_E5.3 | SOT Condition 5 Equilibrium Trial 3 |
| SOT_E5_Com | SOT Condition 5 Equilibirum Mean |
| SOT_S5.1 | SOT Condition 5 Strategy Trial 1 |
| SOT_S5.2 | SOT Condition 5 Strategy Trial 2 |
| SOT_S5.3 | SOT Condition 5 Strategy Trial 3 |
| SOT_S5_Com | SOT Condition 5 Strategy Mean |
| SOT_Fro5.1 | SOT Condition 5 COG Frontal plane Trial 1 |
| SOT_Fro5.2 | SOT Condition 5 COG Frontal plane Trial 2 |
| SOT_Fro5.3 | SOT Condition 5 COG Frontal plane Trial 3 |
| SOT_COG5_Fro_Com | SOT Condition 5 COG Frontal plane Mean |
| SOT_Sag5.1 | SOT Condtion 5 COG Sagittal plane Trial 1 |
| SOT_Sag5.2 | SOT Condition 5 COG Sagittal plane Trial 2 |
| SOT_Sag5.3 | SOT Condition 5 COG Sagittal plane Trial 3 |
| SOT_COG5_Sag_Com | SOT Condition 5 COG Sagittal plane Mean |
| SOT_E6.1 | SOT Condition 6 Equilibrium Trial 1 |
| SOT_E6.2 | SOT Condition 6 Equilibrium Trial 2 |
| SOT_E6.3 | SOT Condition 6 Equilibrium Trial 3 |
| SOT_E6_Com | SOT Condition 6 Equilibirium Mean |
| SOT_S6.1 | SOT Condition 6 Strategy Trial 1 |
| SOT_S6.2 | SOT Condition 6 Strategy Trial 2 |
| SOT_S6.3 | SOT Condition 6 Strategy Trial 3 |
| SOT_S6_Com | SOT Condition 6 Strategy Mean |
| SOT_Fro6.1 | SOT Condition 6 COG Frontal plane Trial 1 |
| SOT_Fro6.2 | SOT Condition 6 COG Frontal plane Trial 2 |
| SOT_Fro6.3 | SOT Condition 6 COG Frontal plane Trial 3 |
| SOT_COG6_Fro_Com | SOT Condition 6 COG Frontal plane Mean |
| SOT_Sag6.1 | SOT Condtion 6 COG Sagittal plane Trial 1 |
| SOT_Sag6.2 | SOT Condition 6 COG Sagittal plane Trial 2 |
| SOT_Sag6.3 | SOT Condition 6 COG Sagittal plane Trial 3 |
| SOT_COG6_Sag_Com | SOT Condition 6 COG Sagittal plane Mean |
| LOS_RT_Composite | LOS Reaction Time Composite in sec |
| LOS_RT_F | LOS Reaction Time anterior in sec |
| LOS_RT_RF | LOS Reaction Time right-anterior in sec |
| LOS_RT_R | LOS Reaction Time right in sec |
| LOS_RT_RB | LOS Reaction Time right-posterior in sec |
| LOS_RT_B | LOS Reaction Time posterior in sec |
| LOS_RT_LB | LOS Reaction Time posterior-left in sec |
| LOS_RT_L | LOS Reaction Time left in sec |
| LOS_RT_LF | LOS Reaction Time anterior-left in sec |
| LOS_MVL_Composite | LOS Movement Velocity Composite in deg/sec |
| LOS_MVL_F | LOS Movement Velocity anterior in deg/sec |
| LOS_MVL_RF | LOS Movement Velocity anterior-right in deg/sec |
| LOS_MVL_R | LOS Movement Velocity right in deg/sec |
| LOS_MVL_RB | LOS Movement Velocity posterior-right in deg/sec |
| LOS_MVL_B | LOS Movement Velocity posterior in deg/sec |
| LOS_MVL_BL | LOS Movement Velocity posterior-left in deg/sec |
| LOS_MVL_L | LOS Movement Velocity left in deg/sec |
| LOS_MVL_LF | LOS Movement Velocity anterior-left in deg/sec |
| LOS_DCL_Composite | LOS Directional Control Composite in % |
| LOS_DCL_F | LOS Directional Control anterior in % |
| LOS_DCL_RF | LOS Directional Control anterior-right in % |
| LOS_DCL_R | LOS Directional Control right in % |
| LOS_DCL_RB | LOS Directional Control posterior-right in % |
| LOS_DCL_B | LOS Directional Control posterior in % |
| LOS_DCL_LB | LOS Directional Control posterior-left in % |
| LOS_DCL_L | LOS Directional Control left in % |
| LOS_DCL_LF | LOS Directional Control anterior-left in % |
| LOS_EPE_Comp | LOS Endpoint Excursion Composite in % |
| LOS_EPE_F | LOS Endpoint Excursion anterior in % |
| LOS_EPE_RF | LOS Endpoint Excursion anterior-right in % |
| LOS_EPE_R | LOS Endpoint Excursion right in % |
| LOS_EPE_RB | LOS Endpoint Excursion hinen-right in % |
| LOS_EPE_B | LOS Endpoint Excursion posterior in % |
| LOS_EPE_LB | LOS Endpoint Excursion posterior-left in % |
| LOS_EPE_L | LOS Endpoint Excursion left in % |
| LOS_EPE_LF | LOS Endpoint Excursion anterior-left in % |
| LOS_MXE_Comp | LOS Maximal Excursion Composite in % |
| LOS_MXE_F | LOS Maximal Excursion anterior in % |
| LOS_MXE_RF | LOS Maximal Excursion anterior right in % |
| LOS_MXE_R | LOS Maximal Excursion right in % |
| LOS_MXE_RB | LOS Maximal Excursion posterior-right in % |
| LOS_MXE_B | LOS Maximal Excursion posterior in % |
| LOS_MXE_LB | LOS Maximal Excursion posterior-left in % |
| LOS_MXE_L | LOS Maximal Excursion left in % |
| LOS_MXE_LF | LOS Maximal Excursion anterior-left in % |
| MCT_Latency_Composite | MCT Latency Composite in msec |
| MCT_SMALL_Back_li | MCT Latency small Range of motion posterior left in msec |
| MCT_SMALL_Back_re | MCT Latency small Range of motion posterior right in msec |
| MCT_SMALL_Back_Wei | MCT Weight small Range of motion posterior |
| MCT_SMALL_Back_Str | MCT Strength of Symmetry small Range of motion posterior |
| MCT_SMALL_BACK_Amp_li | MCT Amplitude Scaling small Range of motion posterior left |
| MCT_SMALL_BACK_Amp_re | MCT Amplitude Scaling small Range of motion posterior right |
| MCT_MEDIUM_Back_li | MCT Latency medium Range of motion posterior left in msec |
| MCT_MEDIUM_Back_re | MCT Latency medium Range of motion posterior right in msec |
| MCT_MEDIUM_Back_Weig | MCT Weight Symmetry medium Range of motion posterior |
| MCT_MEDIUM_Back_Sym | MCT Strength of Symmetry medium Range of motion posterior |
| MCT_MEDIUM_Back_Amp_li | MCT Amplitude-Scaling medium Range of motion posterior left |
| MCT_MEDIUM_BACK_Amp_re | MCT Amplitude-Scaling medium Range of motion posterior right |
| MCT_LARGE_Back_li | MCT Latency big Range of motion posterior left in msec |
| MCT_LARGE_Back_re | MCT Latency big Range of motion posterior right in msec |
| MCT_LARGE_Back_Wei | MCT Weight Symmetry big Range of motion posterior |
| MCT_LARGE_Back_Str | MCT Strength of Symmetry big Range of motion posterior |
| MCT_LARGE_Back_Amp_li | MCT Amplitude-Scaling big Range of motion posterior left |
| MCT_LARGE_Back_Amp_re | MCT Amplitude-Scaling big Range of motion posterior right |
| MCT_SMALL_FOR_li | MCT Latency small Range of motion anterior left in msec |
| MCT_SMALL_FOR_re | MCT Latency small Range of motion anterior right in msec |
| MCT_SMALL_FOR_Wei | MCT Weight Symmetry small Range of motion anterior |
| MCT_SMALL_FOR_Str | MCT Strength of Symmetry small Range of motion anterior |
| MCT_SMALL_FOR_Amp_li | MCT Amplitude-Scaling small Range of motion anterior |
| MCT_SMALL_FOR_Amp_re | MCT Amplitude-Scaling small Range of motion anterior |
| MCT_MEDIUM_FOR_li | MCT Latency medium Range of motion anterior left in msec |
| MCT_MEDIUM_FOR_re | MCT Latency medium Range of motion anterior right in msec |
| MCT_MEDIUM_FOR_Wei | MCT Weight Symmetry medium Range of motion anterior |
| MCT_MEDIUM_FOR_Str | MCT Strength of Symmetry medium Range of motion anterior |
| MCT_MEDIUM_FOR_Amp_li | MCT Amplitude-Scaling medium Range of motion anterior left |
| MCT_MEDIUM_FOR_Amp_re | MCT Amplitude-Scaling medium Range of motion anterior right |
| MCT_LARGE_FOR_li | MCT Latency big Range of motion anterior left in msec |
| MCT_LARGE_FOR_re | MCT Latency big Range of motion anterior right in msec |
| MCT_LARGE_FOR_Wei | MCT Weight Symmetry big Range of motion anterior |
| MCT_LARGE_FOR_Str | MCT Strength of Symmetry big Range of motion anterior |
| MCT_LARGE_FOR_Amp_li | MCT Amplitude-Scaling big Range of motion anterior left |
| MCT_LARGE_FOR_Amp_re | MCT Amplitude-Scaling big Range of motion anterior right |
| Gruppe_Gesamtindex_Sport | Gruppeneinteilung Gesamtindex Sport nach MET-Stunden/Woche, definiert nach Tabelle 6 des Leitfadens zur Anwendung und Auswertung des MoMo-AFB aus 2016 |
| Subgruppen_Sport | Einteilung der Subgruppen nach der Aktivitätsnorm (Gruppeneinteilung Gesamtindex Sport, definiert nach Tabelle 6 des Leitfadens zur Anwendung und Auswertung des MoMo-AFB aus 2016) |
| Aktivität_ges | Gesamtaktivität Tage pro Woche mit mind. 60 min moderate bis intensive Aktivität |
| Aktivität_alter_min | Aktivität im altersspezifischen Setting in Minuten/Woche |
| Aktivität_alter_MET | Aktivität im altersspezifischen Setting in MET-Minuten/Woche, MET-Werte definiert nach Tabelle 7 und 8 des Leitfadens zur Anwendung und Auswertung des MoMo-AFB aus 2016 |
| Aktivität_alter_METStunden | Aktivität im altersspezifischen Setting in MET-Stunden/Woche, MET-Werte definiert nach Tabelle 7 und 8 des Leitfadens zur Anwendung und Auswertung des MoMo-AFB aus 2016 |
| Aktivität_alltag_min | Alltagsaktivität in Minuten/Woche |
| Aktivität_alltag_MET | Alltagsaktivität in MET-Minuten/Woche, MET-Werte definiert nach Tabelle 7 und 8 des Leitfadens zur Anwendung und Auswertung des MoMo-AFB aus 2016 |
| Aktivität_alltag_METStunden | Alltagsaktivität in MET-Stunden/Woche, MET-Werte definiert nach Tabelle 7 und 8 des Leitfadens zur Anwendung und Auswertung des MoMo-AFB aus 2016 |
| Sportverein | Mitgliedschaft im Sportverein oder Teilnahme am Wettkampfsport |
| Aktivität_verein_min | Körperliche Sportliche Aktivität im Verein in Minuten/Woche |
| Aktivität_verein_MET | Körperliche Sportliche Aktivität im Verein in MET-Minuten/Woche, MET-Werte definiert nach Tabelle 7 und 8 des Leitfadens zur Anwendung und Auswertung des MoMo-AFB aus 2016 |
| Aktivität_verein_METStunden | Körperliche Sportliche Aktivität im Verein in MET-Stunden/Woche, MET-Werte definiert nach Tabelle 7 und 8 des Leitfadens zur Anwendung und Auswertung des MoMo-AFB aus 2016 |
| Aktivität_freizeit_min | Körperliche-sportliche Aktivität außerhalb des Vereins in Minuten/Woche |
| Aktivität_freizeit_MET | Körperliche-sportliche Aktivität außerhalb des Vereins in MET-Minuten/Woche, MET-Werte definiert nach Tabelle 7 und 8 des Leitfadens zur Anwendung und Auswertung des MoMo-AFB aus 2016 |
| Aktivität_freizeit_METStunden | Körperliche-sportliche Aktivität außerhalb des Vereins in MET-Stunden/Woche, MET-Werte definiert nach Tabelle 7 und 8 des Leitfadens zur Anwendung und Auswertung des MoMo-AFB aus 2016 |
| Aktivität_gesSport_min | Gesamtaktivität der körperlich-sportlichen Aktivität in Minuten/Woche |
| Aktivität_gesSport_MET | Gesamtaktivität im Sport in MET-Minuten/Woche, MET-Werte definiert nach Tabelle 7 und 8 des Leitfadens zur Anwendung und Auswertung des MoMo-AFB aus 2016 |
| Aktivität_gesSport_METStunden | Gesamtaktivität im Sport in MET-Stunden/Woche, MET-Werte definiert nach Tabelle 7 und 8 des Leitfadens zur Anwendung und Auswertung des MoMo-AFB aus 2016 |
| Minutenbew_ges | Gesamte Bewegung in Minuten |
| MET_ges | gesamte MET-Minuten/Woche, MET-Werte definiert nach Tabelle 7 und 8 des Leitfadens zur Anwendung und Auswertung des MoMo-AFB aus 2016 |
| MET_ges_Stunden | gesamte MET-Stunden/Woche, MET-Werte definiert nach Tabelle 7 und 8 des Leitfadens zur Anwendung und Auswertung des MoMo-AFB aus 2016 |
| I1_Ausfüller | 1 Wer füllt den Fragebogen aus? |
| I3_Aktivität_7tage | 3 An wie vielen der letzten sieben Tage warst du für mindestens 60 Minuten am Tag körperlich aktiv? in Tagen |
| I4_Aktivität_woche | 4 An wie vielen Tagen einer normalen Woche bist du für mindestens 60 Minuten am Tag körperlich aktiv? in Tagen |
| I5_Interesse_Sport | 5 Wie groß ist dein Intresse an Sport? |
| I6_Schwimmen | 6 Kannst du schwimmen? |
| I7_Schwimmen_Alter | 7 Wenn ja, wie alt warst du, als du schwimmen gelernt hast? Alter in Zahlen |
| II8_Schulart | 8 Welche Schulart besuchst du? |
| II9_Ganztagsschule | 9 Ist das eine Ganztagsschule? |
| II10_Schule_nachmittags | 10 An wie vielen Tagen einer normalen Woche bist du nachmittags an der Schule? |
| II11_Schulverletzungen | 11 Wie viele Unfälle, Verletzungen oder Vergiftungen, die vom Arzt behandelt werden mussten, hattest du in den letzten 12 Monaten in der Schule? Anzahl |
| II12_Unfallorte | 12 Wo traten diese Unfälle, Verletzungen oder Vergiftungen auf? (Mehrfachnennung möglich) |
| II13a_Mathe | 13a Welche Note hattest du im letzten Zeugnis in Mathematik? in Noten |
| II13b_Deutsch | 13b Welche Note hattest du im letzten Zeugnis in Deutsch? in Noten |
| II13c_Sport | 13c Welche Note hattest du im letzten Zeugnis Sport? in Noten |
| II14_Sportunterricht_anzahl | 14 An wie vielen Tagen einer normalen Woche hast du regulären Sportunterricht in der Schule? |
| II15_Sportunterricht_minuten | 15 Wie viele Minuten regulären Sportunterricht hast du in einer normalen Woche insgesamt? in Minuten |
| II16_Anstrengung_sportunterricht | 16 Wie sehr strengst du dich dabei in der Regeln an? |
| II17_Sportangebote_schule | 17 Gibt es an deiner Schule Sportangebote? |
| II18_Sportangebote_teilnahme | 18 Besuchst du mindestens ein Sportangebot (z. B. Sport-AG) in der Schule außerhalb des regulären Sportunterrichts? |
| II19_Sportangebote_art | 19 An welchen Sportangebot(en) nimmst du teil? Codierung siehe Anhangstabelle 8 MET-Äquivalent des Leitfadens zur Anwendung und Auswertung des MoMo-AFB aus 2016 |
| II20_Sportangebote_min | 20 Wie viele Minuten betreibst du dieses/diese Sportangebot(e) in der Woche? in Minuten |
| II21_Sportangebote_anstrengung | 21 Wie sehr strengst du dich dabei in der Regel an? |
| II22a_Schulweg | 22a Wie kommst du meistens zur Schule? |
| II22b_Schulweg | 22b Wie viele Minuten brauchst du für eine Strecke ohne Rückweg? in Minuten |
| III23_Gartenarbeit_tage | 23 An wie vielen Tagen pro Woche arbeitest du im Garten oder in der Landwirtschaft mit (z. B. Kirschen pflücken, Unkraut jäten, Hof kehren)? in Tagen |
| III24_Gartenarbeit_protag | 24 Wie viele Minuten arbeitest du durchschnittlich an einem dieser Tage im Garten oder in der Landwirtschaft mit? in Minuten |
| III25_Gartenarbeit_anstrengung | 25 Wie sehr strengst du dich dabei an? |
| III26_Haushalt_tage | 26 An wie vielen Tagen pro Woche arbeitest du im Haushalt mit (z.B. Staubsaugen, Putzen)? in Tagen |
| III27_Haushalt_minprotag | 27 Wie viele Minuten arbeitest du durchschnittlich an einem dieser Tage im Haushalt mit? in Minuten |
| III28_Haushalt_anstrengung | 28 Wie sehr strengst du dich dabei an? |
| III29a_Laufstrecke_km | 29a Wie groß ist die Entfernung, die du täglich zu Fuß gehst (nur Wegstrecken, kein Laufsport)? in km |
| III29b_Laufstrecke_min | 29b Wie groß ist die Entfernung, die du täglich zu Fuß gehst (nur Wegstrecken, kein Laufsport)? in min |
| III30_Laufstrecke_anstrengung | 30 Wie sehr strengst du dich dabei an? |
| III31a_Fahrrad_km | 31 a Wie groß ist die Entfernung, die du täglich mit dem Fahrrad (kein Radsport) oder anderen unmotorisierten Beförderungsmitteln (z. B. Longboard) fährst? in km |
| III31b_Fahrrad_min | 31 b Wie groß ist die Entfernung, die du täglich mit dem Fahrrad (kein Radsport) oder anderen unmotorisierten Beförderungsmitteln (z. B. Longboard) fährst? in min |
| III32_Fahrrad_anstrengung | 32 Wie sehr strengst du dich dabei an? |
| III33_Spielen_draußen_tage | 33 Wie häufig spielst du pro Woche in der Regel im Freien (z.B. Fangen spielen, Gummitwist)? in Tagen |
| III34_Spielen_draußen_protag | 34 Wie lange spielst du an diesen Tagen durchschnittlich im Freien? in Minuten |
| III35_Spielen_draußen_anstrengung | 35 Wie sehr strengst du dich dabei an? |
| IV36_Vereinsmitglied | 36 Bist du Mitglied im Verein? |
| IV37a1_Verein_sporart | 37a1 Welche Sportart betreibst du im Verein? Codierung siehe Anhangstabelle 8 MET-Äquivalent des Leitfadens zur Anwendung und Auswertung des MoMo-AFB aus 2016 |
| IV37a2_Verein_dauer | 37a2 Wie lange betreibst du die jeweilige Sportart insgesamt pro Woche (ohne Wegzeit, Umziehen, Duschen)? in Minuten |
| IV37a3_Verein_monate | 37a3 In welchen Monaten führst du die jeweilige Sportart aus? Angabe in Anzahl der Monate |
| Verein_Jahresindex1 | Anzahl der Monate pro Jahr, in denen Sport betrieben wird |
| IV37a4_Verein_anstrengung | 37a4 Wie sehr strengst du dich dabei an? Aktivitätsklassifikation, siehe Anhangstabelle 7 MET-Werte nach Aktivitätsklassifikation des Leitfadens zur Anwednung und Auswertung des MoMo-AFB aus 2016 |
| IV37a5_Verein_Wettkampf | Ich bin in dieser Sportart Wettkampfsportler. |
| IV37b1_Verein_sporart | 37b1 Welche Sportart betreibst du im Verein? Codierung siehe Anhangstabelle 8 MET-Äquivalent des Leitfadens zur Anwendung und Auswertung des MoMo-AFB aus 2016 |
| IV37b2_Verein_dauer | 37b2 Wie lange betreibst du die jeweilige Sportart insgesamt pro Woche (ohne Wegzeit, Umziehen, Duschen)? in Minuten |
| IV37b3_Verein_monate | 37b3 In welchen Monaten führst du die jeweilige Sportart aus? Angabe in Anzahl der Monate |
| Verein_Jahresindex2 | Anzahl der Monate pro Jahr, in denen Sport betrieben wird |
| IV37b4_Verein_anstrengung | 37b4 Wie sehr strengst du dich dabei an? Aktivitätsklassifikation, siehe Anhangstabelle 7 MET-Werte nach Aktivitätsklassifikation des Leitfadens zur Anwednung und Auswertung des MoMo-AFB aus 2016 |
| IV37b5_Verein_Wettkampf1 | Ich bin in dieser Sportart Wettkampfsportler. |
| IV37c1_Verein_sporart | 37c1 Welche Sportart betreibst du im Verein? Codierung siehe Anhangstabelle 8 MET-Äquivalent des Leitfadens zur Anwendung und Auswertung des MoMo-AFB aus 2016 |
| IV37c2_Verein_dauer | 37c2 Wie lange betreibst du die jeweilige Sportart insgesamt pro Woche (ohne Wegzeit, Umziehen, Duschen)? in Minuten |
| IV37c3_Verein_monate | 37c3 In welchen Monaten führst du die jeweilige Sportart aus? Angabe in Anzahl der Monate |
| Verein_Jahresindex3 | Anzahl der Monate pro Jahr, in denen Sport betrieben wird |
| IV37c4_anstrengung | 37c4 Wie sehr strengst du dich dabei an? Aktivitätsklassifikation, siehe Anhangstabelle 7 MET-Werte nach Aktivitätsklassifikation des Leitfadens zur Anwednung und Auswertung des MoMo-AFB aus 2016 |
| IV37c5_Verein_Wettkampf1 | Ich bin in dieser Sportart Wettkampfsportler. |
| IV37d1_Verein_sporart | 37d1 Welche Sportart betreibst du im Verein? Codierung siehe Anhangstabelle 8 MET-Äquivalent des Leitfadens zur Anwendung und Auswertung des MoMo-AFB aus 2016 |
| IV37d2_Verein_dauer | 37d2 Wie lange betreibst du die jeweilige Sportart insgesamt pro Woche (ohne Wegzeit, Umziehen, Duschen)? in Minuten |
| IV37d3_Verein_monate | 37d3 In welchen Monaten führst du die jeweilige Sportart aus? Angabe in Anzahl der Monate |
| Verein_Jahresindex4 | Anzahl der Monate pro Jahr, in denen Sport betrieben wird |
| IV37d4_Verein_anstrengung | 37d4 Wie sehr strengst du dich dabei an? Aktivitätsklassifikation, siehe Anhangstabelle 7 MET-Werte nach Aktivitätsklassifikation des Leitfadens zur Anwednung und Auswertung des MoMo-AFB aus 2016 |
| IV37d5_Verein_Wettkampf1 | Ich bin in dieser Sportart Wettkampfsportler. |
| V38_Freizeitsport | Betreibst du eine Sportart(en) außerhalb des Vereins und der Schule? |
| V39_Freizeitsport_organisation | In welcher Organisationsform betreibst du die Sportart(en)? |
| V40a1_Freizeit_sportart | Welche Sportart betreibst du außerhalb des Vereins? Codierung siehe Anhangstabelle 8 MET-Äquivalent des Leitfadens zur Anwendung und Auswertung des MoMo-AFB aus 2016 |
| V40a2_Freizeit_dauer | Wie lange betreibst du die jeweilige Sportart insgesamt pro Woche (ohne Wegzeit, Umziehen, Duschen)? in Minuten |
| V40a3_Freizeit_monate | In welchen Monaten führst du die jeweilige Sportart aus? Angabe in Anzahl der Monate |
| Freizeit_Jahresindex1 | Anzahl der Monate pro Jahr, in denen Sport betrieben wird |
| V40a4_Freizeit_anstrengung | 40a4 Wie sehr strengst du dich dabei an? Aktivitätsklassifikation, siehe Anhangstabelle 7 MET-Werte nach Aktivitätsklassifikation des Leitfadens zur Anwednung und Auswertung des MoMo-AFB aus 2016 |
| V40a5_Freizeit_Wettkampf | Ich bin in dieser Sportart Wettkampfsportler. |
| V40b1_Freizeit_sportart | Welche Sportart betreibst du außerhalb des Vereins? Codierung siehe Anhangstabelle 8 MET-Äquivalent des Leitfadens zur Anwendung und Auswertung des MoMo-AFB aus 2016 |
| V40b2_Freizeit_dauer | Wie lange betreibst du die jeweilige Sportart insgesamt pro Woche (ohne Wegzeit, Umziehen, Duschen)? in Minuten |
| V40b3_Freizeit_monate | In welchen Monaten führst du die jeweilige Sportart aus? Angabe in Anzahl der Monate |
| Freizeit_Jahresindex2 | Anzahl der Monate pro Jahr, in denen Sport betrieben wird |
| V40b4_Freizeit_anstrengung | 40b4 Wie sehr strengst du dich dabei an? Aktivitätsklassifikation, siehe Anhangstabelle 7 MET-Werte nach Aktivitätsklassifikation des Leitfadens zur Anwednung und Auswertung des MoMo-AFB aus 2016 |
| V40b5_Freizeit_Wettkampf1 | Ich bin in dieser Sportart Wettkampfsportler. |
| V40c1_Freizeit_sportart | Welche Sportart betreibst du außerhalb des Vereins? Codierung siehe Anhangstabelle 8 MET-Äquivalent des Leitfadens zur Anwendung und Auswertung des MoMo-AFB aus 2016 |
| V40c2_Freizeit_dauer | Wie lange betreibst du die jeweilige Sportart insgesamt pro Woche (ohne Wegzeit, Umziehen, Duschen)? in Minuten |
| V40c3_Freizeit_monate | In welchen Monaten führst du die jeweilige Sportart aus? Angabe in Anzahl der Monate |
| Freizeit_Jahresindex3 | Anzahl der Monate pro Jahr, in denen Sport betrieben wird |
| V40c4_Freizeit_anstrengung | 40c4 Wie sehr strengst du dich dabei an? Aktivitätsklassifikation, siehe Anhangstabelle 7 MET-Werte nach Aktivitätsklassifikation des Leitfadens zur Anwednung und Auswertung des MoMo-AFB aus 2016 |
| V40c5_Freizeit_Wettkampf1 | Ich bin in dieser Sportart Wettkampfsportler. |
| V40d1_Freizeit_sportart | Welche Sportart betreibst du außerhalb des Vereins? Codierung siehe Anhangstabelle 8 MET-Äquivalent des Leitfadens zur Anwendung und Auswertung des MoMo-AFB aus 2016 |
| V40d2_Freizeit_dauer | Wie lange betreibst du die jeweilige Sportart insgesamt pro Woche (ohne Wegzeit, Umziehen, Duschen)? in Minuten |
| V40d3_Freizeit_monate | In welchen Monaten führst du die jeweilige Sportart aus? Angabe in Anzahl der Monate |
| Freizeit_Jahresindex4 | Anzahl der Monate pro Jahr, in denen Sport betrieben wird |
| V40d4_Freizeit_anstrengung | 40d4 Wie sehr strengst du dich dabei an? Aktivitätsklassifikation, siehe Anhangstabelle 7 MET-Werte nach Aktivitätsklassifikation des Leitfadens zur Anwednung und Auswertung des MoMo-AFB aus 2016 |
| V40d5_Freizeit_Wettkampf1 | Ich bin in dieser Sportart Wettkampfsportler. |

**Supplementary Material S2**

**Exploratory factor analysis**

Exploratory factor analysis (EFA) was conducted on 12 indicators of physical activity using maximum likelihood estimation with oblimin rotation. Sampling adequacy was evaluated using the Kaiser-Meyer-Olkin measure and Bartlett's test of sphericity (eTable2, eTable3). The number of factors was determined based on parallel analysis, eigenvalues, and interpretability of the factor solution. Factor scores were computed using the regression method and used in subsequent analysis. **eTable 4** to **eTable 7** summarize EFA results.

**Elastic net regression**

Elastic net regression with 10-fold cross-validation was applied as a data-driven approach for exploratory predictor selection. Both main effects and interaction terms between BMI categories and physical activity measures were specified. Results from the λmin solution are reported in the main manuscript, whereas the more parsimonious λ1se solution is presented in the Supplement (**eTable 8**). Predictors with non-zero coefficients were retained for subsequent regression analyses.

**Linear regression models**

**Model diagnostics of linear regression models**

Model assumptions were evaluated for all linear regression models. Linearity and homoscedasticity were assessed via visual inspection of residuals versus fitted values and the Breusch-Pagan test. Normality of residuals was examined using normal Q-Q plots. Multicollinearity was assessed using variance inflation factors (VIF). For models including categorical predictors and interaction terms, generalized variance inflation factors adjusted for degrees of freedom GVIF(1/(2Df)) were used. Where heteroscedasticity was detected, heteroscedasticity-consistent standard errors (HC3) were applied. Diagnostic plots are shown for representative models (eFigure1, eFigure 2), while numerical diagnostics are reported for all models in eTable 9.

Predictors identified via elastic net regression were entered into multiple linear regression models to examine associations with postural stability outcomes. All models were adjusted for age, sex, body height, BMI category, and physical (PE) grade. Both additive (main-effects-only) models and interaction models including BMI category were estimated. Variables were entered in their original metrics, except for PE grade, which was z-standardized prior to model estimation. Extracted factor scores were mean-centered before inclusion in the regression models. Model fit indices of linear regression models can be found in **eTable 10**. All linear regression results can be found in **eTable 11**.

**Exploratory structural equation modelling**

Structural equation modeling (SEM) was used to provide an integrated overview of associations between demographic factors, physical activity variables, and multiple postural stability outcomes. Prior to model estimation, reaction time was recoded so that higher values consistently indicated better performance across all postural stability measures. Body mass index (BMI) category was dummy-coded with normal weight as the reference category. Continuous predictors were retained in their original metric to facilitate interpretation of main effects. To reduce multicollinearity in interaction terms, continuous predictors involved in interactions with BMI category were mean-centered prior to computation of product terms. Interaction effects were specified between BMI category and selected physical activity variables based on results from the elastic net and linear regression analyses.

An initial SEM including all relevant predictors and interaction terms was specified. To reduce model complexity and mitigate overfitting, the model was subsequently simplified in a stepwise, theory- and data-informed manner, guided by statistical significance, model fit indices, and consistency with results from the linear regression analyses. Correlated residuals between postural stability outcomes were freely estimated to account for shared variance not explained by the predictors.

Models were estimated using robust maximum likelihood (MLR). Model fit was evaluated using standard fit indices, including the chi-square test, comparative fit index (CFI), Tucker-Lewis index (TLI), root mean square error of approximation (RMSEA) with confidence intervals, standardized root mean square residual (SRMR), and information criteria (AIC and BIC). Statistical significance was determined using a threshold of *P* < .05. Regression coefficients are reported in **eTable 12**. Fit indices of the SEM are reported in **eTable 13**.

**Supplementary Material S3**

**Exploratory factor analysis results**

Sampling adequacy was marginal, with a Kaiser-Meyer-Olkin value of 0.58. However, Bartlett's test of sphericity was highly significant, supporting the use of exploratory factor analysis. Inspection of item-level measure of sampling adequacy values indicated that most variables were acceptable, although some items showed marginal sampling adequacy.

**Linear regression models results**

For all linear regression models, standard diagnostic procedures were applied. Linearity and homoscedasticity were assessed using residuals versus fitted value plots, and normality of residuals was evaluated using Q-Q plots. Multicollinearity was examined using variance inflation factors (VIFs), with values <5 considered acceptable. Homoscedasticity was formally tested using the Breusch-Pagan test. For models in which heteroscedasticity was detected, robust standard errors (HC3) were used for statistical inference.
