# Supplementary material for: Physical activity and anthropometric factors as predictors for postural stability in children
Source: Sci Rep. 2026 May 27;16:16425. doi: 10.1038/s41598-026-55265-7 (PMC13216329; doi:10.1038/s41598-026-55265-7)
Supplement: Supplementary file 2 — Supplementary Material 2 [file 41598_2026_55265_MOESM2_ESM.docx]

**Supplementary Figures**

Saskia Brummer, Simon Flock, Anna-Marie Berelsmann, Martin Scholten, Christian Dobel, Orlando Guntinas-Lichius

**Physical activity and anthropometric factors as predictors for postural stability in children**

**Supplementary Figure S1**. Exemplar Regression Diagnostic Plots for the Additive Linear Regression Model Predicting DCL

**Supplementary Figure S2**. Exemplar Regression Diagnostic Plots for the Interaction Model Predicting SOT S5.

This supplemental material has been provided by the authors to give readers additional information about their work.

**Supplementary Figure S1.** Exemplar Regression Diagnostic Plots For the Additive Linear Regression Model Predicting DCL.


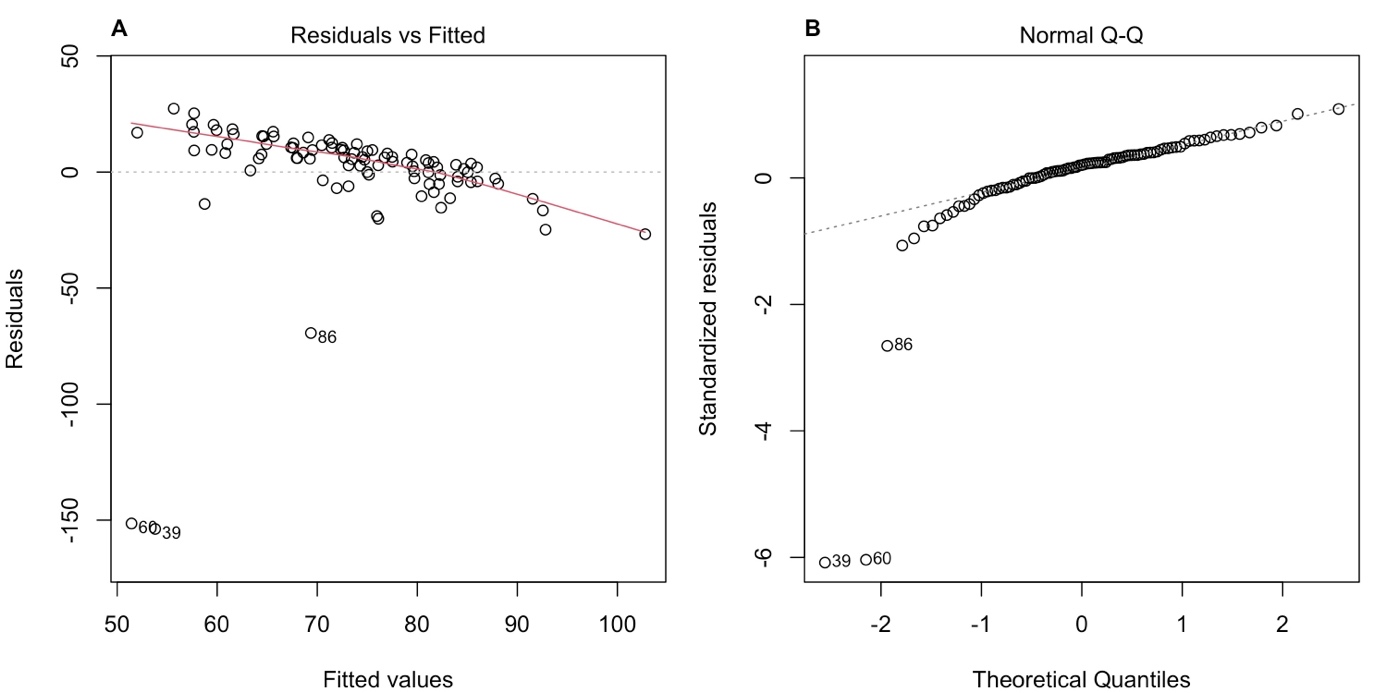


**Notes.** The panel A shows residuals versus fitted values to assess linearity and homoscedasticity. Panel B shows the normal Q-Q plot to assess normailty of residuals.

**Supplementary Figure S2.** Exemplar Regression Diagnostic Plots for the Interaction Model Predicting SOT S5.

*
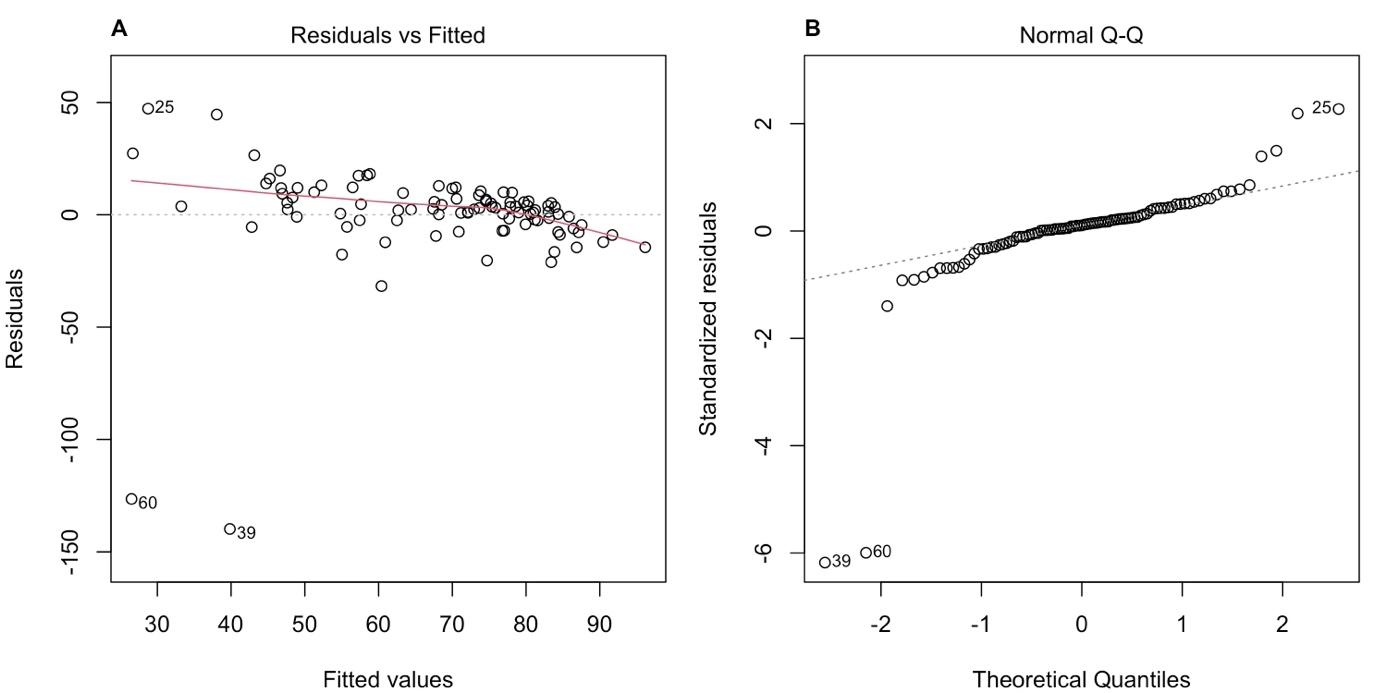
*

**Notes.** Panel A shows residuals versus fitted values to assess linearity and homoscedasticity. Panel B shows the normal Q-Q plot to assess normality of residuals.
