# Supplementary material for: Physical activity and anthropometric factors as predictors for postural stability in children
Source: Sci Rep. 2026 May 27;16:16425. doi: 10.1038/s41598-026-55265-7 (PMC13216329; doi:10.1038/s41598-026-55265-7)
Supplement: Supplementary file 3 — Supplementary Material 3 [file 41598_2026_55265_MOESM3_ESM.docx]

**Supplementary Tables**

Saskia Brummer, Simon Flock, Anna-Marie Berelsmann, Martin Scholten, Christian Dobel, Orlando Guntinas-Lichius

**Physical activity and anthropometric factors as predictors for postural stability in children**

**Supplementary Table S1**. Sample Characteristics by BMI Category

**Supplementary Table S2**. Kaiser-Meyer-Olkin Measure and Bartlett's Test of Sphericity

**Supplementary Table S3.** Kaiser-Meyer-Olkin (KMO) Measure for Individual Variables

**Supplementary Table S4**. Factor Loadings from Exploratory Factor Analysis

**Supplementary Table S5**. Model Fit Indices of EFA

**Supplementary Table S6**. Item-to-Factor Assignment from Exploratory Factor Analysis

**Supplementary Table S7**. Extracted Factor Scores by BMI Category

**Supplementary Table S8**. Predictors of Postural Stability Outcomes Identified by Elastic Net Regression

**Supplementary Table S9**. Homoscedasticity and Multicollinearity Diagnostics for Linear Regression Models.

**Supplementary Table S10**. Model Fit Indices for Linear Regression Models

**Supplementary Table** **S11**. Linear Regression Coefficients of Models Predicting Postural Stability OOtcomes

**Supplementary Table 12**. Structural Equation Model Regression Paths for Postural Stability Outcomes

**Supplementary Table 13**. Structural Equation Model Fit Indices

This supplemental material has been provided by the authors to give readers additional information about their work.

## **Supplementary Table S1.** Sample Characteristics by BMI Category

|  | **Overall**  (n = 95)^1^ | **Normal weight**  (n = 51)^1^ | **Overweight**  (n = 12)^1^ | **Obesity**  (n = 32)^1^ |
| --- | --- | --- | --- | --- |
| **Age** in years**, mean (SD)** | 13.03 (2.64) | 13.25 (2.79) | 13.83 (2.52) | 12.38 (2.34) |
| Female Sex, n (%) | 51/95 (54%) | 28/51 (55%) | 8/12 (67%) | 15/32 (47%) |
| Male Sex, n (%) | 44/95 (46%) | 23/51 (45%) | 4/12 (33%) | 17/32 (53%) |
| Height in cm, mean (SD) | 161.19 (13.38) | 160.19 (13.81) | 163.77 (12.16) | 161.81 (13.36) |
| Weight in kg, mean (SD) | 64.32 (22.34) | 50.99 (13.55) | 67.45 (12.14) | 84.39 (21.27) |
| BMI^a^, mean (SD) | 24.28 (6.51) | 19.43 (2.67) | 24.93 (1.79) | 31.77 (4.36) |
| Activity, mean (SD) |  |  |  |  |
| Activity Daily (MET)^b^ | 47.16 (46.28) | 39.42 (34.62) | 56.67 (46.77) | 55.93 (59.78) |
| Club Activity (MET)^b^ | 17.65 (26.97) | 23.53 (30.15) | 13.25 (27.29) | 9.92 (18.68) |
| Leisure-Time Activity (MET)^b^ | 10.73 (22.11) | 11.36 (23.61) | 9.10 (13.38) | 10.34 (22.75) |
| Physical Education Grade | 1.74 (1.08) | 1.39 (0.98) | 2.18 (0.58) | 2.15 (1.19) |
| ^1^Data are presented as mean (SD) for continuous variables and No./total No. (%) for categorical variables.  ^a^BMI indicates body mass index; categories defined according to German reference percentiles for children.^23^  ^b^MET indicates metabolic equivalent of task; values were derived from published activity classifications. ^24^  ^c^German 6-point grading system ranging from 1 to 6. The lower the grade, the better it is: a “1” is an excellent grade, whereas “5 and “6” are fail grades. | | | | |

## **Supplementary Table S2.** Kaiser-Meyer-Olkin Measure and Bartlett's Test of Sphericity

| **Index** | **Value** |
| --- | --- |
| KMO (overall) | 0.583 |
| Bartlett χ^2^ (df) | 628.585 (66) |
| Bartlett *P* value | < .001 |
| N (items) | 12 |
| N (observations) | 95 |

## **Notes.** KMO indicates sampling adequacy. Bartlett test of sphericity confirms correlation matrix deviates from identity (*P* < .001).

## **Supplementary Table S3.** Kaiser-Meyer-Olkin (KMO) Measure for Individual Variables

| **Variable** | **MSA** |
| --- | --- |
| Weekly Activity | 0.776 |
| Interest in Sport | 0.727 |
| Leisure-Time Activity (MET) | 0.664 |
| Daily Activity (MET) | 0.660 |
| Club Activity (MET) | 0.585 |
| Leisure-Time Competitions | 0.584 |
| Playing | 0.581 |
| Cycling (km/day) | 0.570 |
| Cycling (min/day) | 0.526 |
| Walking (min/day) | 0.516 |
| Club Competitions | 0.506 |
| Walking (km/day) | 0.494 |

**Notes.** The table reports the individual measures of sampling adequacy (MSA) for each variable included in the exploratory factor analysis. Higher values indicate greater shared variance with other variables and better suitability for factor analysis. Values ≥ .50 are generally considered acceptable. **Abbreviations.** MET, metabolic equivalent of task.

**Supplementary Table S4.** Factor Loadings from Exploratory Factor Analysis

| **Item** | **ML4** | **ML3** | **ML2** | **ML5** | **ML1** |
| --- | --- | --- | --- | --- | --- |
| Walking (min/day) | -0.01 | 0.00 | 0.98 | 0.01 | 0.06 |
| Walking (km/day) | 0.03 | -0.01 | 0.90 | 0.00 | -0.07 |
| Playing | 0.93 | -0.05 | -0.11 | 0.00 | 0.06 |
| Cycling (km/day) | 0.07 | 0.91 | 0.01 | 0.00 | -0.01 |
| Cycling (min/day) | -0.05 | 0.98 | -0.02 | 0.00 | 0.01 |
| Daily Activity (MET) | 0.91 | 0.08 | 0.14 | -0.02 | -0.04 |
| Leisure-Time Activity (MET) | 0.02 | -0.01 | 0.01 | 0.00 | 0.99 |
| Club Activity (MET) | -0.02 | -0.04 | 0.00 | 0.83 | -0.02 |
| Weekly Activity | 0.39 | -0.07 | 0.05 | 0.10 | 0.12 |
| Interest in Sport | 0.27 | 0.10 | -0.02 | 0.53 | 0.11 |
| Leisure-Time Competitions | -0.12 | 0.18 | 0.03 | -0.06 | 0.44 |
| Club Competitions | -0.05 | 0.01 | 0.01 | 0.84 | -0.01 |

**Notes.** Factor loadings are shown for the exploratory factor analysis. Items are assigned to factors based on their highest loading**. Abbreviations.** ML = maximum likelihood factor; MET = metabolic equivalent of task.

**Supplementary Table S5.** Model Fit Indices of EFA

| **Index** | **Value** |
| --- | --- |
| χ^2^ (df) | 20.840 (16) |
| *P* value | 0.185 |
| RMSEA (90% CI) | 0.055 (0.000-0.118) |
| RMSR | 0.019 |
| BIC | -52.020 |

## **Notes.** Model fit indices are reported for the exploratory factor analysis. Non-significant χ^2^ values indicate acceptable model fit. Lower RMSEA and RMSR values indicate better fit. **Abbreviations.** RMSEA, root mean square error of approximation; RMSR, root mean square residual; BIC, Bayesian information criterion.

**Supplementary Table S6.** Item-to-Factor Assignment from Exploratory Factor Analysis

| **Item** | **Primary Factor Label** | **Primary Loading** | **Secondary Loading** | **Cross Loading** |
| --- | --- | --- | --- | --- |
| Club Competitions | Cycling | 0.84 | -0.05 | No |
| Club Activity (MET) | Cycling | 0.83 | -0.04 | No |
| Interest in Sport | Cycling | 0.53 | 0.27 | No |
| Cycling (min/day) | Daily Activity | 0.98 | -0.05 | No |
| Cycling (km/day) | Daily Activity | 0.91 | 0.07 | No |
| Walking (min/day) | Leisure Time Activity | 0.98 | 0.06 | No |
| Walking (km/day) | Leisure Time Activity | 0.90 | -0.07 | No |
| Leisure-Time Activity (MET) | Sports Club Activity | 0.99 | 0.02 | No |
| Leisure-Time Competitions | Sports Club Activity | 0.44 | 0.18 | No |
| Playing | Walking / Playing | 0.93 | -0.11 | No |
| Daily Activity (MET) | Walking / Playing | 0.91 | 0.14 | No |
| Weekly Activity | Walking / Playing | 0.39 | 0.12 | No |

**Notes.** Items were assigned to factors according to their primary loading (i.e., the highest absolute loading), which is presented with the corresponding factor to illustrate factor composition. Secondary loadings and indications of cross-loadings (Yes/No) are included to show the extend of multiple factor associations. Factor scores for subsequent analyses were computed from the final EFA solution using the regression method and merges with the analytic dataset.

## **Supplementary Table S7.** Extracted Factor Scores^a^ by BMI Category^b^

|  | **Overall**  (n = 95)^1^ | **Normal weight**^b^  (n = 51)^1^ | **Overweight**^b^  (n = 12)^1^ | **Obesity**^b^ (n = 32)^1^ |
| --- | --- | --- | --- | --- |
| Factor Score, mean (SD) |  |  |  |  |
| **Walking** | 0.00 (0.97) | -0.16 (0.74) | 0.10 (0.87) | 0.21 (1.27) |
| **Daily Activity** | 0.00 (0.98) | -0.09 (0.99) | 0.38 (0.88) | -0.01 (1.00) |
| **Leisure**-Time **Activity** | 0.00 (0.98) | 0.02 (0.99) | 0.40 (0.73) | -0.18 (1.01) |
| **Cycling** | 0.00 (0.92) | 0.26 (1.03) | -0.21 (0.74) | -0.34 (0.64) |
| **Sports Club Activity** | 0.00 (1.00) | 0.03 (1.06) | -0.08 (0.61) | -0.02 (1.03) |
| ^1^Data are presented as mean (SD). Standardized factor scores (mean = 0, SD =1) derived using the regression method.  ^b^ body mass index categories are defined according to German reference percentiles for children.^23^ | | | | |

**Supplementary Table S8.** Predictors of PPostural SStability OOutcomes IIdentified by Elastic Net Regression

|  | **Predictor** | **Estimate** | λ**Type** | **Class** |
| --- | --- | --- | --- | --- |
| DCL (%) | Body Height, cm | 0.103 | λmin | Main effect |
| EPE (%) | Body Height, cm | -0.179 | λmin | Main effect |
|  | Body Height, cm | 0.000 | λ1se | Main effect |
| MVL (deg/sec) | PE Grade | 0.000 | λmin | Main effect |
|  | PE Grade | 0.000 | λ1se | Main effect |
| MXE (%) | Daily Activity | -3.994 | λmin | Main effect |
|  | PE Grade | -3.081 | λmin | Main effect |
|  | Cycling | 1.397 | λmin | Main effect |
|  | Body Height, cm | -0.229 | λmin | Main effect |
|  | Age | 0.036 | λmin | Main effect |
|  | Body Height, cm | 0.000 | λ1se | Main effect |
| MCT (deg/sec) | Age | -9.053 | λmin | Main effect |
|  | Walking | 2.888 | λmin | Main effect |
|  | Leisure-Time Activity | 2.479 | λmin | Main effect |
|  | Cycling | 1.265 | λmin | Main effect |
|  | Body Height, cm | 0.685 | λmin | Main effect |
|  | Female Sex | -0.440 | λmin | Main effect |
|  | PE Grade | 0.313 | λmin | Main effect |
|  | Male Sex | 0.183 | λmin | Main effect |
|  | Obesity (BMI Category) | -1.766 | λmin | Interaction |
|  | PE Grade * Overweight (BMI Category) | -42.320 | λmin | Interaction |
|  | Club Activity * Obesity (BMI Category) | -30.399 | λmin | Interaction |
|  | Female Sex * Obesity (BMI Category) | -20.225 | λmin | Interaction |
|  | Daily Activity * Overweight (BMI Category) | -17.817 | λmin | Interaction |
|  | Walking * Overweight (BMI Category) | -17.804 | λmin | Interaction |
|  | Leisure-Time Activity * Overweight (BMI Category) | 13.883 | λmin | Interaction |
|  | Daily Activity * Obesity (BMI Category) | 13.433 | λmin | Interaction |
|  | Cycling * Obesity (BMI Category) | 12.778 | λmin | Interaction |
|  | Female Sex * Overweight (BMI Category) | 11.890 | λmin | Interaction |
|  | Walking * Obesity (BMI Category) | -11.091 | λmin | Interaction |
|  | PE Grade * Obesity (BMI Category) | -8.532 | λmin | Interaction |
| SOT Composite (%) | Body Height, cm | 0.282 | λmin | Main effect |
|  | Body Height, cm | 0.000 | λ1se | Main effect |
| SOT E1(%) | Age | 1.861 | λmin | Main effect |
|  | Age | 1.026 | λ1se | Main effect |
|  | Cycling | -0.451 | λmin | Main effect |
|  | Female Sex | 0.305 | λmin | Main effect |
|  | Male Sex | -0.294 | λmin | Main effect |
|  | PE Grade | -0.117 | λmin | Main effect |
|  | Body Height, cm | 0.084 | λ1se | Main effect |
|  | Cycling | -0.045 | λ1se | Main effect |
|  | Body Height, cm | 0.036 | λmin | Main effect |
|  | Walking * Obesity (BMI Category) | -0.953 | λmin | Interaction |
|  | Walking * Obesity (BMI Category) | -0.319 | λ1se | Interaction |
|  | Club Activity * Obesity (BMI Category) | 0.279 | λmin | Interaction |
| SOT E2 (%) | Age | 1.644 | λmin | Main effect |
|  | Female Sex | 0.261 | λmin | Main effect |
|  | Male Sex | -0.259 | λmin | Main effect |
|  | Cycling | -0.172 | λmin | Main effect |
|  | Body Height, cm | 0.060 | λ1se | Main effect |
|  | Body Height, cm | 0.030 | λmin | Main effect |
|  | Leisure-Time Activity | 0.008 | λmin | Main effect |
|  | Obesity (BMI Category) | -0.152 | λmin | Interaction |
|  | Walking * Obesity (BMI Category) | -0.714 | λmin | Interaction |
| SOT E3 (%) | Age | 1.236 | λmin | Main effect |
|  | Walking | -1.164 | λmin | Main effect |
|  | Leisure-Time Activity | 0.649 | λmin | Main effect |
|  | Cycling | -0.556 | λmin | Main effect |
|  | Daily Activity | 0.537 | λmin | Main effect |
|  | Club Activity | -0.230 | λmin | Main effect |
|  | Body Height, cm | 0.033 | λmin | Main effect |
|  | PE Grade | 0.014 | λmin | Main effect |
|  | Body Height, cm | 0.000 | λ1se | Main effect |
|  | Club Activity * Obesity (BMI Category) | -0.821 | λmin | Interaction |
|  | Walking * Obesity (BMI Category) | -0.519 | λmin | Interaction |
|  | Leisure-Time Activity * Obesity (BMI Category) | 0.000 | λmin | Interaction |
| SOT E4 (%) | Daily Activity | -0.900 | λmin | Main effect |
|  | Walking | -0.718 | λmin | Main effect |
|  | Body Height, cm | 0.317 | λmin | Main effect |
|  | Body Height, cm | 0.000 | λ1se | Main effect |
| SOT E5 (%) | Body Height, cm | 0.362 | λmin | Main effect |
|  | Body Height, cm | 0.000 | λ1se | Main effect |
| SOT E6 (%) | Body Height, cm | 0.448 | λmin | Main effect |
|  | Body Height, cm | 0.000 | λ1se | Main effect |
| SOT S1 (%) | Body Height, cm | 0.012 | λmin | Main effect |
|  | Body Height, cm | 0.000 | λ1se | Main effect |
| SOT S2 (%) | Body Height, cm | 0.001 | λmin | Main effect |
|  | Body Height, cm | 0.000 | λ1se | Main effect |
| SOT S4 (%) | Daily Activity | -0.627 | λmin | Main effect |
|  | Walking | -0.617 | λmin | Main effect |
|  | Body Height, cm | -0.096 | λmin | Main effect |
| SOT S5 (%) | Age | 7.122 | λmin | Main effect |
|  | PE Grade | -4.026 | λmin | Main effect |
|  | Club Activity | 0.932 | λmin | Main effect |
|  | Body Height, cm | -0.548 | λmin | Main effect |
|  | Daily Activity | -0.459 | λmin | Main effect |
|  | Leisure-Time Activity | 0.158 | λmin | Main effect |
|  | Obesity (BMI Category) | -8.451 | λmin | Interaction |
|  | Daily Activity * Overweight (BMI Category) | -6.115 | λmin | Interaction |
|  | Leisure-Time Activity * Obesity (BMI Category) | 4.609 | λmin | Interaction |
|  | PE Grade * Overweight (BMI Category) | -1.707 | λmin | Interaction |
| SOT S6 (%) | Age | 5.701 | λmin | Main effect |
|  | PE Grade | -5.594 | λmin | Main effect |
|  | Daily Activity | -0.501 | λmin | Main effect |
|  | Body Height, cm | -0.345 | λmin | Main effect |
|  | Leisure-Time Activity | 0.029 | λmin | Main effect |
|  | Obesity (BMI Category) | -5.874 | λmin | Interaction |
|  | Daily Activity * Overweight (BMI Category) | -0.828 | λmin | Interaction |
|  | Leisure-Time Activity * Obesity (BMI Category) | 0.438 | λmin | Interaction |

**Notes.** Results are shown for the λmin model and the λ1se model of the elastic net regression. All predictors per outcome are shown, for both models with and without interaction terms. Coefficients reflect standardized predictors. **Abbreviations.** DCL, Directional Control; EPE, Endpoint Excursion; MVL, Movement Velocity; MXE, Maximum Excursion; RT, Reaction Time; MCT, Motor Control Test Latency; SOT, Sensory Organization Test; SOT E5, Sensory Organization Test Equilibrium Score, Condition 5; SOT E6 = Sensory Organization Test Equilibrium Score, Condition 6; SOT S5, Sensory Organization Test Strategy Score, Condition 5; SOT S6, Sensory Organization Test Strategy Score, Condition 6; PE, Physical Education

**BMI categories** (normal weight, overweight, obesity) were defined according to German reference percentiles.^23^

**Supplementary Table S9.** Homoscedasticity and Multicollinearity Diagnostics for Linear Regression Models.

| **Outcome** | **Model Type** | **N** | **BP *P*-value** | **Homoscedasticity** | **Max VIF** |
| --- | --- | --- | --- | --- | --- |
| DCL | Additive | 95 | 0.087 | Yes | 1.72 |
| EPE | Additive | 95 | 0.080 | Yes | 1.72 |
| MCT | Additive | 95 | 0.909 | Yes | 1.76 |
| MCT | Interaction | 95 | 0.987 | Yes | 1.84 |
| MVL | Additive | 95 | 0.073 | Yes | 1.72 |
| MXE | Additive | 95 | 0.110 | Yes | 1.76 |
| RT | Additive | 95 | 0.076 | Yes | 1.72 |
| SOT Composite | Additive | 95 | 0.081 | Yes | 1.72 |
| SOT E5 | Additive | 95 | 0.072 | Yes | 1.72 |
| SOT E6 | Additive | 95 | 0.117 | Yes | 1.72 |
| SOT S5 | Additive | 95 | 0.118 | Yes | 1.73 |
| SOT S5 | Interaction | 95 | 0.032 | No | 1.82 |
| SOT S6 | Additive | 95 | 0.108 | Yes | 1.76 |
| SOT S6 | Interaction | 95 | 0.025 | No | 1.82 |

**Notes.** Homoscedasticity was assessed using the Breusch Pagan test, and multicollinearity was evaluated using variance inflation factors. Adjusted GVIF values were used for models including categorical predictors and interactions.

**Abbreviations.** BP, Breusch-Pagan; VIF, variance inflation factor; DCL, Directional Control; EPE, Endpoint Excursion; MVL, Movement Velocity; MXE, Maximum Excursion; RT, Reaction Time; MCT, Motor Control Test Latency; SOT, Sensory Organization Test; SOT E5, Sensory Organization Test Equilibrium Score, Condition 5; SOT E6, Sensory Organization Test Equilibrium Score, Condition 6; SOT S5, Sensory Organization Test Strategy Score, Condition 5; SOT S6, Sensory Organization Test Strategy Score, Condition 6.

## **Supplementary Table S10.** Model Fit Indices for Linear Regression Models

| **Outcome** | **Model** | **N** | **R²** | **Adj R²** | **F** | **Model df** | **Residual df** |
| --- | --- | --- | --- | --- | --- | --- | --- |
| LOS DCL | Additive | 95 | 0.132 | 0.073 | 2.23 | 6 | 88 |
| LOS EPE | Additive | 95 | 0.163 | 0.106 | 2.85 | 6 | 88 |
| LOS MVL | Additive | 95 | 0.109 | 0.048 | 1.79 | 6 | 88 |
| LOS MXE | Additive | 95 | 0.233 | 0.162 | 3.27 | 8 | 86 |
| LOS RT | Additive | 95 | 0.110 | 0.049 | 1.81 | 6 | 88 |
| MCT Latency | Additive | 95 | 0.124 | 0.042 | 1.52 | 8 | 86 |
| MCT Latency | Interaction | 95 | 0.294 | 0.181 | 2.60 | 13 | 81 |
| SOT | Additive | 95 | 0.141 | 0.083 | 2.41 | 6 | 88 |
| SOT E5 | Additive | 95 | 0.144 | 0.086 | 2.47 | 6 | 88 |
| SOT E6 | Additive | 95 | 0.170 | 0.114 | 3.01 | 6 | 88 |
| SOT S5 | Additive | 95 | 0.257 | 0.197 | 4.30 | 7 | 87 |
| SOT S5 | Interaction | 95 | 0.331 | 0.233 | 3.37 | 12 | 82 |
| SOT S6 | Additive | 95 | 0.259 | 0.200 | 4.35 | 7 | 87 |
| SOT S6 | Interaction | 95 | 0.323 | 0.224 | 3.26 | 12 | 82 |

**Notes.** Model fit indices are shown separately for additive models (main effects only) and interaction models (including selected BMI × activity interactions). All models controlled for age, sex, body height, BMI category, and physical education grade.

**Abbreviations.** LOS, Limits of Stability; SOT, Sensory Organization Test; MCT, Motor Control Test.

## **Supplementary Table S11.** Linear Regression Coefficients of Models Predicting Postural Stability Outcomes

| **Model** | **SE Type** | **Predictor** | **Est (95% CI)** | **β (95% CI)** | ***P* value** |
| --- | --- | --- | --- | --- | --- |
| DCL Additive | Classical | Age (y) | 3.90 (0.34, 7.47) | 0.37 (0.03, 0.71) | 0.032 |
|  |  | BMI (Obesity) | -1.03 (-14.64, 12.57) | -0.04 (-0.53, 0.45) | 0.881 |
|  |  | BMI (Overweight) | -9.98 (-27.67, 7.70) | -0.36 (-1.00, 0.28) | 0.265 |
|  |  | Body height (cm) | -0.30 (-0.94, 0.34) | -0.15 (-0.46, 0.16) | 0.352 |
|  |  | PE grade | -6.78 (-12.99, -0.57) | -0.24 (-0.47, -0.02) | 0.033 |
|  |  | Sex (female) | 1.48 (-10.32, 13.28) | 0.05 (-0.37, 0.48) | 0.804 |
| EPE Additive | Classical | Age (y) | 2.88 (-0.59, 6.35) | 0.28 (-0.06, 0.61) | 0.103 |
|  |  | BMI (Obesity) | -8.48 (-21.73, 4.77) | -0.31 (-0.79, 0.17) | 0.207 |
|  |  | BMI (Overweight) | -17.88 (-35.10, -0.66) | -0.65 (-1.27, -0.02) | 0.042 |
|  |  | Body height (cm) | -0.50 (-1.12, 0.13) | -0.24 (-0.55, 0.06) | 0.118 |
|  |  | PE Grade (z) | -5.81 (-11.86, 0.24) | -0.21 (-0.43, 0.01) | 0.060 |
|  |  | Sex (female) | -3.60 (-15.09, 7.90) | -0.13 (-0.55, 0.29) | 0.536 |
| MCT Additive | Classical | Age (y) | -3.84 (-10.19, 2.50) | -0.21 (-0.57, 0.14) | 0.232 |
|  |  | BMI (Obesity) | -11.08 (-35.09, 12.94) | -0.23 (-0.74, 0.27) | 0.362 |
|  |  | BMI (Overweight) | -3.01 (-34.24, 28.23) | -0.06 (-0.72, 0.60) | 0.849 |
|  |  | Body Height (cm) | 0.72 (-0.40, 1.83) | 0.20 (-0.11, 0.52) | 0.204 |
|  |  | PE Grade (z) | -10.00 (-20.91, 0.91) | -0.21 (-0.44, 0.02) | 0.072 |
|  |  | Sex (Female) | -7.27 (-28.74, 14.19) | -0.15 (-0.61, 0.30) | 0.502 |
|  |  | Leisure Activity | 8.28 (-2.52, 19.08) | 0.17 (-0.05, 0.39) | 0.131 |
|  |  | Walking | -7.73 (-19.59, 4.14) | -0.16 (-0.40, 0.08) | 0.199 |
| MCT Interaction | Classical | Age (y) | -2.84 (-8.76, 3.07) | -0.16 (-0.49, 0.17) | 0.342 |
|  |  | BMI (Obesity) | -7.61 (-36.79, 21.56) | -0.16 (-0.78, 0.45) | 0.605 |
|  |  | Club x Obesity | -30.88 (-51.39, -10.38) | -0.65 (-1.08, -0.22) | 0.004 |
|  |  | PE Grade x Obesity | -9.33 (-29.98, 11.31) | -0.20 (-0.63, 0.24) | 0.371 |
|  |  | BMI (Overweight) | -21.61 (-82.48, 39.26) | -0.46 (-1.74, 0.83) | 0.482 |
|  |  | Club x Overweight | -11.55 (-57.18, 34.07) | -0.24 (-1.20, 0.72) | 0.616 |
|  |  | PE Grade x Overweight | -41.97 (-97.96, 14.02) | -0.89 (-2.07, 0.30) | 0.140 |
|  |  | Body Height (cm) | 0.71 (-0.35, 1.77) | 0.20 (-0.10, 0.50) | 0.184 |
|  |  | PE grade (z) | 0.91 (-13.40, 15.21) | 0.02 (-0.28, 0.32) | 0.900 |
|  |  | Sex (female) | -4.37 (-30.54, 21.79) | -0.09 (-0.64, 0.46) | 0.740 |
|  |  | club activity | 2.15 (-10.11, 14.41) | 0.05 (-0.21, 0.30) | 0.728 |
|  |  | Sex (Female) x Obesity | -24.65 (-66.08, 16.78) | -0.52 (-1.39, 0.35) | 0.240 |
|  |  | Sex (Female) x Overweight | 40.50 (-23.87, 104.88) | 0.85 (-0.50, 2.21) | 0.214 |
| MVL Additive | Classical | Age (y) | 1.63 (-0.35, 3.60) | 0.28 (-0.06, 0.63) | 0.105 |
|  |  | BMI (Obesity) | 0.31 (-7.21, 7.84) | 0.02 (-0.48, 0.52) | 0.934 |
|  |  | BMI (Overweight) | -6.48 (-16.26, 3.30) | -0.43 (-1.07, 0.22) | 0.192 |
|  |  | Body height (cm) | -0.17 (-0.52, 0.19) | -0.15 (-0.46, 0.17) | 0.354 |
|  |  | PE grade (z) | -3.97 (-7.41, -0.54) | -0.26 (-0.49, -0.04) | 0.024 |
|  |  | Sex (Female) | -1.86 (-8.39, 4.67) | -0.12 (-0.55, 0.31) | 0.573 |
| MXE Additive | Classical | Age (y) | 2.74 (-0.97, 6.44) | 0.24 (-0.09, 0.57) | 0.146 |
|  |  | BMI (Obesity) | -10.17 (-24.46, 4.12) | -0.34 (-0.83, 0.14) | 0.161 |
|  |  | BMI (Overweight) | -14.61 (-32.78, 3.56) | -0.49 (-1.11, 0.12) | 0.114 |
|  |  | Body Height (cm) | -0.53 (-1.20, 0.14) | -0.24 (-0.54, 0.07) | 0.123 |
|  |  | PE Grade (z) | -5.04 (-11.49, 1.41) | -0.17 (-0.39, 0.05) | 0.124 |
|  |  | Sex (Female) | -5.77 (-18.47, 6.93) | -0.20 (-0.62, 0.23) | 0.369 |
|  |  | Cycling | 2.34 (-4.39, 9.07) | 0.07 (-0.14, 0.28) | 0.492 |
|  |  | Daily Activity | -6.58 (-12.77, -0.39) | -0.22 (-0.42, -0.01) | 0.038 |
| RT Additive | Classical | Age (y) | 1.65 (-0.24, 3.55) | 0.30 (-0.04, 0.64) | 0.086 |
|  |  | BMI (Obesity) | 1.44 (-5.78, 8.65) | 0.10 (-0.40, 0.60) | 0.693 |
|  |  | BMI (Overweight) | -5.70 (-15.08, 3.68) | -0.39 (-1.04, 0.25) | 0.230 |
|  |  | Body Height (cm) | -0.16 (-0.50, 0.18) | -0.15 (-0.46, 0.17) | 0.357 |
|  |  | PE Grade (z) | -4.02 (-7.31, -0.72) | -0.28 (-0.50, -0.05) | 0.017 |
|  |  | Sex (Female) | -1.19 (-7.44, 5.07) | -0.08 (-0.51, 0.35) | 0.708 |
| SOT Composite Additive | Classical | Age (y) | 3.59 (0.11, 7.07) | 0.35 (0.01, 0.68) | 0.044 |
|  |  | BMI (Obesity) | -0.05 (-13.33, 13.22) | -0.00 (-0.49, 0.49) | 0.994 |
|  |  | BMI (Overweight) | -10.05 (-27.31, 7.20) | -0.37 (-1.00, 0.26) | 0.250 |
|  |  | Body Height (cm) | -0.09 (-0.72, 0.53) | -0.04 (-0.35, 0.26) | 0.772 |
|  |  | PE Grade (z) | -6.32 (-12.38, -0.25) | -0.23 (-0.45, -0.01) | 0.041 |
|  |  | Sex (Female) | 2.32 (-9.19, 13.84) | 0.09 (-0.34, 0.51) | 0.689 |
| SOT E5 Additive | Classical | Age (y) | 3.34 (-0.13, 6.81) | 0.32 (-0.01, 0.66) | 0.059 |
|  |  | BMI (Obesity) | 0.22 (-13.01, 13.44) | 0.01 (-0.48, 0.49) | 0.974 |
|  |  | BMI (Overweight) | -9.91 (-27.09, 7.28) | -0.36 (-1.00, 0.27) | 0.255 |
|  |  | Body Height (cm) | -0.01 (-0.63, 0.61) | -0.01 (-0.31, 0.30) | 0.972 |
|  |  | PE Grade (z) | -5.21 (-11.25, 0.83) | -0.19 (-0.41, 0.03) | 0.090 |
|  |  | Sex (Female) | 4.94 (-6.53, 16.41) | 0.18 (-0.24, 0.60) | 0.395 |
| SOT E6 Additive | Classical | Age (y) | 3.50 (-0.07, 7.06) | 0.32 (-0.01, 0.66) | 0.054 |
|  |  | BMI (Obesity) | -1.26 (-14.85, 12.33) | -0.04 (-0.52, 0.43) | 0.854 |
|  |  | BMI (Overweight) | -7.60 (-25.27, 10.06) | -0.27 (-0.89, 0.35) | 0.395 |
|  |  | Body Height (cm) | 0.07 (-0.57, 0.72) | 0.03 (-0.27, 0.34) | 0.819 |
|  |  | PE Grade (z) | -6.47 (-12.68, -0.26) | -0.23 (-0.45, -0.01) | 0.041 |
|  |  | Sex (Female) | 4.94 (-6.85, 16.73) | 0.17 (-0.24, 0.59) | 0.407 |
| SOT S5 Additive | Classical | Age (y) | 3.55 (0.18, 6.92) | 0.33 (0.02, 0.65) | 0.039 |
|  |  | BMI (Obesity) | -17.20 (-30.09, -4.32) | -0.62 (-1.08, -0.15) | 0.009 |
|  |  | BMI (Overweight) | -14.95 (-31.55, 1.65) | -0.53 (-1.13, 0.06) | 0.077 |
|  |  | Body Height (cm) | -0.64 (-1.24, -0.03) | -0.31 (-0.59, -0.02) | 0.039 |
|  |  | PE Grade (z) | -4.88 (-10.84, 1.08) | -0.17 (-0.39, 0.04) | 0.107 |
|  |  | Sex (Female) | 5.22 (-6.15, 16.60) | 0.19 (-0.22, 0.59) | 0.364 |
|  |  | Club Activity | 2.84 (-2.68, 8.36) | 0.10 (-0.10, 0.30) | 0.309 |
| SOT S5 Interaction | HC3 | Age (y) | 2.87 (-0.60, 6.34) | 0.27 (-0.13, 0.67) | 0.104 |
|  |  | BMI (Obesity) | -17.87 (-30.59, -5.15) | -0.64 (-1.02, -0.26) | 0.006 |
|  |  | Daily x Obesity | -4.77 (-17.51, 7.97) | -0.17 (-0.82, 0.49) | 0.459 |
|  |  | Leisure x Obesity | 10.96 (-1.55, 23.48) | 0.38 (-0.41, 1.17) | 0.085 |
|  |  | BMI (Overweight) | -11.40 (-31.35, 8.55) | -0.41 (-1.19, 0.38) | 0.259 |
|  |  | Daily x Overweight | -20.24 (-39.08, -1.40) | -0.71 (-2.44, 1.02) | 0.036 |
|  |  | Leisure x Overweight | 6.89 (-15.27, 29.04) | 0.24 (-0.78, 1.27) | 0.538 |
|  |  | Body Height (cm) | -0.58 (-1.20, 0.03) | -0.28 (-0.55, -0.01) | 0.062 |
|  |  | PE Grade (z) | -3.13 (-9.06, 2.81) | -0.11 (-0.43, 0.21) | 0.297 |
|  |  | Sex (Female) | 2.63 (-8.51, 13.77) | 0.09 (-0.51, 0.70) | 0.640 |
|  |  | Daily Activity | 1.50 (-5.68, 8.67) | 0.05 (-0.02, 0.12) | 0.679 |
|  |  | Leisure Activity | -0.98 (-8.14, 6.17) | -0.03 (-0.20, 0.13) | 0.785 |
| SOT S6 Additive | Classical | Age (y) | 3.28 (-0.16, 6.73) | 0.31 (-0.02, 0.63) | 0.061 |
|  |  | BMI (Obesity) | -17.11 (-29.98, -4.24) | -0.61 (-1.07, -0.15) | 0.010 |
|  |  | BMI (Overweight) | -12.72 (-29.51, 4.07) | -0.45 (-1.05, 0.14) | 0.136 |
|  |  | Body Height (cm) | -0.48 (-1.10, 0.14) | -0.23 (-0.52, 0.07) | 0.131 |
|  |  | PE Grade (z) | -6.29 (-12.20, -0.37) | -0.22 (-0.43, -0.01) | 0.038 |
|  |  | Sex (Female) | 3.03 (-8.31, 14.38) | 0.11 (-0.30, 0.51) | 0.596 |
|  |  | Daily Activity | -1.09 (-6.83, 4.66) | -0.04 (-0.24, 0.16) | 0.708 |
| SOT S6 Interaction | HC3 | Age (y) | 2.89 (-0.63, 6.40) | 0.27 (-0.14, 0.68) | 0.106 |
|  |  | BMI (Obesity) | -17.21 (-30.08, -4.34) | -0.61 (-0.92, -0.30) | 0.009 |
|  |  | Daily x Obesity | -7.23 (-20.13, 5.66) | -0.25 (-0.87, 0.36) | 0.267 |
|  |  | Leisure x Obesity | 9.32 (-3.34, 21.98) | 0.32 (-0.36, 1.01) | 0.147 |
|  |  | BMI (Overweight) | -8.75 (-28.93, 11.44) | -0.31 (-1.12, 0.50) | 0.391 |
|  |  | Daily x Overweight | -19.78 (-38.84, -0.72) | -0.69 (-2.49, 1.11) | 0.042 |
|  |  | Leisure x Overweight | 5.58 (-16.84, 27.99) | 0.19 (-1.09, 1.48) | 0.622 |
|  |  | Body Height (cm) | -0.44 (-1.06, 0.18) | -0.21 (-0.49, 0.07) | 0.160 |
|  |  | PE Grade (z) | -5.18 (-11.18, 0.83) | -0.18 (-0.51, 0.14) | 0.090 |
|  |  | Sex (Female) | 1.83 (-9.44, 13.10) | 0.07 (-0.53, 0.66) | 0.747 |
|  |  | Daily Activity | 1.45 (-5.81, 8.71) | 0.05 (-0.03, 0.13) | 0.691 |
|  |  | Leisure Activity | -1.12 (-8.36, 6.13) | -0.04 (-0.20, 0.12) | 0.760 |

**Notes.** Unstandardized coefficients with 95% CIs, standardized coefficients (β), and P values are reported. Models include additive (main effects only) and selected interaction terms. All models were adjusted for age, sex, height, BMI category, and physical education grade. All variables were entered in their original units, except PE Grade, which was z-standardized before model estimation. Extracted factor scores were mean-centered prior to analysis. BMI category was included as a categorical predictor with three levels: Normal Weight, Overweight, and Obesity. Heteroscedasticity-consistent (HC3) standard errors were used in models where heteroscedasticity was detected. **Abbreviations.** DCL, Directional Control; EPE, Endpoint Excursion; MVL, Movement Velocity; MXE, Maximum Excursion; RT, Reaction Time; MCT, Motor Control Test Latency; SOT, Sensory Organization Test; SOT E5, Sensory Organization Test Equilibrium Score, Condition 5; SOT E6, Sensory Organization Test Equilibrium Score, Condition 6; SOT S5, Sensory Organization Test Strategy Score, Condition 5; SOT S6, Sensory Organization Test Strategy Score, Condition 6; PE, Physical Education

**BMI categories** (Normal Weight, Overweight, Obesity) were defined according to German reference percentiles.^23^

**Supplementary Table S12.** Structural Equation Model Regression Paths for Postural Stability Outcomes

| **Outcome** | **Predictor** | **Est (95% CI)** | **β** | **P** |
| --- | --- | --- | --- | --- |
| LOS MVL | Age | 0.49 (-0.27, 1.24) | 0.09 | 0.210 |
|  | PE Grade | -0.79 (-1.90, 0.31) | -0.06 | 0.160 |
| LOS RT | Age | 0.57 (-0.15, 1.28) | 0.10 | 0.123 |
|  | PE Grade | -0.91 (-1.91, 0.10) | -0.07 | 0.078 |
| LOS DCL | Age | 2.16 (0.80, 3.51) | 0.21 | 0.002 |
|  | PE Grade | -0.59 (-2.45, 1.26) | -0.02 | 0.530 |
| LOS EPE | Age | 0.30 (-1.11, 1.71) | 0.03 | 0.678 |
|  | Overweight | -3.18 (-6.32, -0.05) | -0.04 | 0.046 |
|  | PE Grade | -0.01 (-1.15, 1.14) | -0.00 | 0.988 |
| LOS MXE | Age | 0.16 (-1.34, 1.67) | 0.01 | 0.830 |
|  | DDaily AActivity | -1.89 (-3.01, -0.77) | -0.06 | <0.001 |
| MCT Latency | Age | -0.84 (-4.59, 2.91) | -0.05 | 0.661 |
|  | Obesity | -20.46 (-40.69, -0.23) | -0.20 | 0.047 |
|  | PE Grade | -4.14 (-12.60, 4.33) | -0.09 | 0.338 |
|  | Club Activity | -6.48 (-15.59, 2.62) | -0.13 | 0.163 |
|  | Club x Obesity | -21.04 (-37.38, -4.70) | -0.25 | 0.012 |
| SOT | Age | 2.82 (1.45, 4.19) | 0.28 | <0.001 |
|  | PE Grade | -1.83 (-3.68, 0.02) | -0.07 | 0.053 |
| SOT E5 | Age | 3.11 (1.53, 4.70) | 0.30 | <0.001 |
|  | PE Grade | -1.65 (-3.68, 0.39) | -0.07 | 0.113 |
| SOT E6 | Age | 3.63 (2.08, 5.18) | 0.34 | <0.001 |
|  | PE Grade | -2.39 (-5.02, 0.23) | -0.09 | 0.074 |
| SOT S5 | Age | 1.53 (0.00, 3.06) | 0.15 | 0.050 |
|  | Obesity | -18.01 (-24.33, -11.69) | -0.32 | <0.001 |
|  | Overweight | -4.45 (-7.94, -0.96) | -0.06 | 0.012 |
|  | Body Height | -0.24 (-0.43, -0.06) | -0.12 | 0.009 |
|  | Daily Activity | -0.40 (-1.51, 0.71) | -0.01 | 0.483 |
|  | Daily x Obesity | 1.19 (-1.84, 4.21) | 0.03 | 0.442 |
|  | Leisure Activity | 0.38 (-0.90, 1.66) | 0.01 | 0.560 |
|  | Leisure x Obesity | 3.77 (-0.41, 7.95) | 0.08 | 0.077 |
| SOT S6 | Age | 1.08 (-0.29, 2.45) | 0.11 | 0.123 |
|  | Overweight | -3.28 (-6.42, -0.15) | -0.04 | 0.040 |
|  | Obesity | -17.05 (-22.82, -11.29) | -0.30 | <0.001 |
|  | PE grade | -1.54 (-3.21, 0.14) | -0.06 | 0.073 |
|  | Daily Activity | -0.47 (-1.78, 0.84) | -0.02 | 0.484 |
|  | Daily x Overweight | 0.38 (-2.77, 3.53) | 0.00 | 0.814 |
|  | Leisure Activity | 0.22 (-1.06, 1.49) | 0.01 | 0.740 |
|  | Leisure x Obesity | 2.30 (-1.29, 5.90) | 0.05 | 0.210 |

**Notes.** Unstandardized (Est) and standardized (β) coefficients, and p values are reported for each predictor-outcome path as specified in the model. Continuous physical activity predictors included in interaction terms were mean-centered; non-interaction predictors and covariates are reported on their original scale. Interactions included where specified: Club x Obesity (Club Activity × Obesity), Daily x Overweight / Daily x Obesity (Daily Activity × BMI Category), and Leisure x Obesity / Leisure x Overweight (Leisure Activity × BMI Category).

**Abbreviations.** LOS RT, Limits of Stability Reaction Time; LOS MVL, Limits of Stability Movement Velocity; LOS MXE, Limits of Stability Maximum Excursion; LOS DCL = Limits of Stability Directional Control; LOS EPE, Limits of Stability Endpoint Excursion; SOT, Sensory Organization Test; SOT E5, Sensory Organization Test Equilibrium Score 5; SOT E6, Sensory Organization Test Equilibrium Score 6; SOT S5, Sensory Organization Test Strategy Score 5; SOT S6, Sensory Organization Test Strategy Score 6; MCT, Motor Control Test latency; PE, Physical Education.

**Supplementary Table S13.** Structural Equation Model Fit Indices

| **Index** | **Value** |
| --- | --- |
| χ^2^ (df) | 297.017 (94) |
| *P* value | <.001 |
| CFI | 0.926 |
| TLI | 0.853 |
| RMSEA (90% CI) | 0.151 (0.132-0.170) |
| SRMR | 0.095 |
| AIC | 7352.16 |
| BIC | 7617.76 |

**Notes.** Fit indices from the final exploratory structural equation model. Lower RMSEA/SRMR and higher CFI/TLI indicate better fit.

**Abbreviations.** χ^2^, chi-square; CFI, comparative fit index; TLI, Tucker–Lewis index; RMSEA, root mean square error of approximation; SRMR, standardized root mean square residual; AIC, Akaike information criterion; BIC, Bayesian information criterion
